# Multi-Strategy Improved Red-Tailed Hawk Algorithm for Real-Environment Unmanned Aerial Vehicle Path Planning

**DOI:** 10.3390/biomimetics10010031

**Published:** 2025-01-06

**Authors:** Mingen Wang, Panliang Yuan, Pengfei Hu, Zhengrong Yang, Shuai Ke, Longliang Huang, Pai Zhang

**Affiliations:** 1 Laboratory for Robot Mobility Localization and Scene Deep Learning Technology, Guizhou Equipment Manufacturing Polytechnic, Guiyang 550025, China; mingw4215@gmail.com (M.W.); wangme2496@gmail.com (P.H.); yang_zheng_rong@163.com (Z.Y.); 18302657238@163.com (S.K.); 18083479298@163.com (L.H.); zl541089269@163.com (P.Z.); 2 State Key Laboratory of Public Big Data, Guizhou University, Guiyang 550025, China

**Keywords:** UAV path planning, intelligent optimization algorithm, red-tailed hawk algorithm, IEEE CEC2017, trust domain

## Abstract

In recent years, unmanned aerial vehicle (UAV) technology has advanced significantly, enabling its widespread use in critical applications such as surveillance, search and rescue, and environmental monitoring. However, planning reliable, safe, and economical paths for UAVs in real-world environments remains a significant challenge. In this paper, we propose a multi-strategy improved red-tailed hawk (IRTH) algorithm for UAV path planning in real environments. First, we enhance the quality of the initial population in the algorithm by using a stochastic reverse learning strategy based on Bernoulli mapping. Then, the quality of the initial population is further improved through a dynamic position update optimization strategy based on stochastic mean fusion, which enhances the exploration capabilities of the algorithm and helps it explore promising solution spaces more effectively. Additionally, we proposed an optimization method for frontier position updates based on a trust domain, which better balances exploration and exploitation. To evaluate the effectiveness of the proposed algorithm, we compare it with 11 other algorithms using the IEEE CEC2017 test set and perform statistical analysis to assess differences. The experimental results demonstrate that the IRTH algorithm yields competitive performance. Finally, to validate its applicability in real-world scenarios, we apply the IRTH algorithm to the UAV path-planning problem in practical environments, achieving improved results and successfully performing path planning for UAVs.

## 1. Introduction

Unmanned aerial vehicle (UAV) technology is of paramount importance at the present time. In the military field, it is a key force in modern warfare. Reconnaissance UAVs can break through high-risk environments to obtain key information, while attack UAVs can realize precision strikes and reduce casualties, greatly changing military strategies and combat modes [1]. In the civil field, UAVs can efficiently obtain high-precision data in geographic surveying and mapping, solving traditional surveying and mapping problems [2]. In environmental monitoring, they can penetrate into complex zones to provide timely and accurate information for environmental protection and resource management. In agricultural production, they can promote the development of precision agriculture and improve yield and quality [3], and they can quickly respond to emergency rescues to rapidly assess the situation of a disaster, search for survivors, and buy time for rescue operations [4].

Path planning, as a key technology for UAVs to perform tasks, is the cornerstone to guarantee the safe, efficient, and reliable operation of UAVs, and provides solid support for the wide application and expansion of UAV technology in various fields. In complex practical application scenarios, whether it is military reconnaissance and strike missions, or actions such as geographic surveying and mapping [5], environmental monitoring [6], agricultural operations [7], and emergency rescue [4] in the civil field, reasonable path planning is the primary prerequisite to ensure that the UAV completes its tasks successfully. It is required in the intelligent navigation of the UAV flight, determining the optimal flight route of the UAV from the starting point to the target point. Accurate path-planning technology enables the UAV to flexibly navigate in an environment full of obstacles, such as when performing tasks in areas with tall buildings in the city, skillfully avoiding all kinds of buildings, avoiding the risk of collision, and guaranteeing flight safety [8]. At the same time, in the face of multiple mission requirements and complex environmental constraints, good path planning can enable the UAV to fly in the most energy-efficient way, effectively extending the endurance time and improving the efficiency of mission execution. In addition, for the concealment needs in military operations [9], path-planning technology can help UAVs choose routes that are not easily detected by the enemy, enhancing the confidentiality and success rate of military operations.

Path-planning algorithms can be mainly classified into two categories: traditional algorithms and intelligent optimization algorithms. The traditional path-planning algorithms mainly include three kinds: Dijkstra’s algorithm [10], A* algorithm [11], and Floyd’s algorithm [12]. Among them, Dijkstra’s algorithm iteratively calculates the shortest paths to other nodes starting from the initial node, it is a deterministic global optimal search but lacks heuristic information to guide it, which may result in excessive computation in large, complex environments [13]. The A star algorithm is an algorithm based on heuristic information, which reduces unnecessary node expansion by guiding the search through a heuristic function [14]. The time complexity is related to the quality of the heuristic function, which degenerates to Dijkstra’s algorithm in the worst case. Therefore, the A star algorithm relies heavily on the definition and consistency of the heuristic function. Floyd’s algorithm is used to find the shortest path between all vertex pairs by considering the intermediate node update information. Although its computational complexity is high, it can handle complex path relationships and is suitable for scenarios that require comprehensive shortest path information.

However, traditional algorithms often struggle with complex terrain, dynamic obstacles, and multiple constraints, while intelligent optimization algorithms can effectively find feasible paths through mechanisms such as the evolution in genetic algorithms and the group collaboration in particle swarm algorithms. The intelligent optimization algorithms mainly include classical genetic algorithms, particle swarm optimization algorithms, and ant colony algorithms, as well as the newly proposed Neural Dynamics Optimization [15], Enterprise Development Optimization [16], and Polar Lights Optimization [17]. In recent years, with the rise of various types of optimization problems, intelligent optimization algorithms have made remarkable advancements. On one hand, classical intelligent optimization algorithms have been continuously improved and refined. For example, on the basis of retaining its core idea of simulating biological evolution, genetic algorithms have improved convergence speed and optimization accuracy by refining genetic operations and adjusting parameter settings, making them more efficient in solving complex optimization problems [18]. The particle swarm optimization algorithm is also constantly exploring new learning strategies and parameter adaptive mechanisms to enhance the performance and adaptability of the algorithm to cope with different types of problems [19]. The ant colony algorithm, on the other hand, has carried out in-depth research on pheromone updating rules and path-searching strategies to further improve the quality and efficiency of solutions [20].

On the other hand, new intelligent optimization algorithms keep emerging. For example, the neural population dynamic optimization algorithm [15], a new type of intelligent optimization algorithm, uses an attractor trend strategy to guide the neural population toward making optimal decisions, ensuring the algorithm’s exploitation ability. It diverges from the neural population and the attractor by coupling with other neural populations, enhancing the algorithm’s exploration ability. Finally, the information projection strategy is used to control communication between the neural populations, facilitating the transition from exploration to exploitation, and offering new ideas and methods for solving complex optimization problems. Moreover, Hashim, Fatma A. et al. proposed the Archimedes optimization algorithm (AOA) based on the Archimedes principle [21]. The algorithm optimizes by simulating the principle of buoyancy force applied upward on an object partially or completely submerged in a fluid, and it is shown through experiments on CEC2017 and four engineering design problems that the AOA algorithm is a high-performance optimization tool for solving complex problems efficiently. Some hybrid intelligent optimization algorithms also combine different approaches, leveraging the strengths of various algorithms, overcoming the shortcomings of a single approach, and demonstrating strong performance in solving complex multimodal and constrained optimization problems. Meanwhile, the application fields of intelligent optimization algorithms are continuously expanding. In logistics, intelligent optimization algorithms are widely used for path planning, warehouse layout optimization, and distribution route optimization to help enterprises improve operational efficiency and reduce costs. In the energy sector, intelligent optimization algorithms are used to optimize integrated energy systems and microgrid scheduling, achieving efficient use and reasonable distribution of energy. Intelligent optimization algorithms also play an increasingly important role in engineering design, machine learning, artificial intelligence, and other fields, providing strong support for solving various complex practical problems. Table 1 summarizes the most recent optimization algorithms.

The RTH algorithm [35], as one of the intelligent optimization algorithms, has been widely used in various fields since it was proposed. Azeddine Houari and his team applied the RTH algorithm to extract parameters for proton exchange membrane fuel cells and tested it on seven real-world engineering problems, benchmarking it against other published algorithms. The experimental results indicated that RTH outperforms most other methods in the majority of cases. Furthermore, to address the issue of conventional MPPT being unable to differentiate between local and global MPP, Almousa, MT, and colleagues introduced a single-sensor global MPPT method based on the RTH algorithm for PV systems connected via DC links, operating under PSCs [36]. This method effectively reduces the number of sensors and decreases the controller’s cost. However, as the NFL theorem suggests that no algorithm performs optimally across all optimization problems, it is essential to refine the algorithm for our specific optimization challenge. Qin, XQ et al. proposed an enhanced RTH algorithm (ERTH) with multiple elite strategies and chaotic mapping for solving multi-cost optimization problems in cloud task scheduling [37]. The ERTH algorithm achieved competitive results through experimental validation.

Based on the above research, this paper proposes a multi-strategy improved RTH algorithm (IRTH). The specific contributions are as follows.

1. A stochastic reverse learning strategy based on Bernoulli mapping is utilized to enhance the quality of the population, allowing the algorithm to explore more promising spaces.
2. The dynamic position update optimization strategy using stochastic mean fusion makes the algorithm less likely to fall into a local optimal solution during exploration and increases the probability that the algorithm will find a globally optimal solution.
3. The convergence speed of the algorithm is improved using a trust domain-based optimization method for frontier position updating, which employs a dynamic trust domain radius to provide a trade-off between convergence speed and accuracy, achieving better performance.
4. The algorithms were qualitatively analyzed using 29 test functions from the IEEE CEC2017 test set and compared with 11 other algorithms to obtain competitive results. Most importantly, the algorithms were statistically analyzed to fully analyze the superior performance of IRTH.
5. The IRTH algorithm is applied to the UAV path-planning problem in a real environment and compared with other comparative algorithms.

The next part of this paper is organized as follows: Section 2 gives a brief introduction to the original RTH algorithm; Section 3 gives a detailed introduction of the trust domain approach and other improvement strategy proposed in this paper; in Section 4, we apply the IRTH algorithm in numerical optimization experiments and analyze the experimental results in detail; in Section 5, we apply the algorithm to the real-environment UAV planning problem and provide a comprehensive analysis of its advantages and disadvantages; in Section 6, we summarize and outlook the work in this paper to clarify the direction of future work.

## 2. Red-Tailed Hawk (RTH) Algorithm

In this section, since the algorithm proposed in this paper is an improvement on the RTH algorithm, we provide a brief description of the RTH algorithm. The RTH algorithm is inspired by the hunting behavior of the red-tailed hawk. During hunting, the red-tailed hawk goes through three phases: high soaring, low soaring, and swooping. The mathematical model of the three parts is as follows:

### 2.1. High-Soaring Stage

During a red-tailed hawk’s hunt, it soars through the sky in order to better find food. This behavior is modeled as shown in Equation (1).

(1) $$X\left(t\right)=X_{best}+\left(X_{mean}-X\left(t-1\right)\right)*Levy\left(dim\right)*TF\left(t\right),$$

where X(t) denotes the current position of the red-tailed hawk, $X_{best}$ indicates the current optimal position, $X_{mean}$ indicates the current average position, Levy(dim) denotes the Levy flight distribution function, which is determined using Equation (2), and TF(t) represents the transition factor function, calculated using Equation (4).

(2) $$Levy\left(\dim\right)=s*\frac{µ*\sigma }{{\left|v\right|}^{\beta -1}},$$

where s and β is a constant, the value is 0.01 and 1.5, respectively. dim is the problem dimension, and µ and v are random numbers belonging to the range [0 1]. σ is calculated according to Equation (3).

(3) $$\sigma =\left(\frac{г\left(1+\beta \right)*\sin\left(\frac{\pi \beta }{2}\right)}{г\left(1+\frac{\beta }{2}\right)*\beta *2^{\left(1-\frac{\beta }{2}\right)}}\right),$$

(4) $$TF\left(t\right)=1+\sin\left(2.5+\left(\frac{t}{T_{max}}\right)\right),$$

where $T_{max}$ denotes the maximum number of iterations.

### 2.2. Low-Soaring Stage

After finding suitable prey through the high-soaring phase, the red-tailed hawk will fly low to lock on to the prey for better hunting. This behavior is modeled as shown in Equation (5).

(5) $$X\left(t\right)=X_{best}+\left(x\left(t\right)+y\left(t\right)\right)*StepSize\left(t\right),$$

where StepSize(t) is calculated according to Equation (6).

(6) $$StepSize\left(t\right)=X\left(t\right)-X_{mean},$$

where x and y represent directional coordinates, computed using Equation (7).

(7) $$\{\begin{array}{c} x\left(t\right)=R\left(t\right)*\sin\left(\theta \left(t\right)\right)\\ y\left(t\right)=R\left(t\right)*\cos\left(\theta \left(t\right)\right) \end{array}\{\begin{array}{c} R\left(t\right)=R_{0}*\left(r-\frac{t}{T_{max}}\right)*rand\\ \theta \left(t\right)=A*\left(1-\frac{t}{T_{max}}\right)*rand \end{array}\{\begin{array}{c} x\left(t\right)=\frac{x\left(t\right)}{\max\left|x\left(t\right)\right|}\\ y\left(t\right)=\frac{y\left(t\right)}{\max\left|y\left(t\right)\right|} \end{array},$$

where $R_{0}$ represents the initial radius value [0.5, 3], A represents the angle gain, the value is [5, 15], rand is a random gain [0, 1], and r is a control gain [1, 2].

### 2.3. Stooping and Swooping Stage

After a high-flying phase and a low-flying phase, the red-tailed hawk locks on to its prey, at which point it needs to hunt. During this phase, the red-tailed hawk will dive at the prey to ensure a kill shot, so this behavior is modeled using Equation (8).

(8) $$X\left(t\right)=\alpha \left(t\right)*X_{best}+x\left(t\right)*StepSize1\left(t\right)+y\left(t\right)*StepSize2\left(t\right)$$

where StepSize1(t) can be calculated according to Equation (9), and StepSize2(t) can be calculated according to Equation (10).

(9) $$StepSize1\left(t\right)=X\left(t\right)-TF\left(t\right)*X_{mean}$$

(10) $$StepSize2\left(t\right)=G\left(t\right)*X\left(t\right)-TF\left(t\right)*X_{best}$$

where α and G represent the acceleration and gravity factors, respectively, and they can be determined using Equations (11) and (12).

(11) $$\alpha \left(t\right)=sin^{2}\left(2.5-\frac{t}{T_{max}}\right)$$

(12) $$G\left(t\right)=2*\left(1-\frac{t}{T_{max}}\right)$$

where α represents the hawk’s acceleration, which grows with increasing t to enhance convergence speed, while G signifies the gravitational effect that weakens as the hawk approaches the prey, thereby reducing exploitation diversity. The pseudocode of the RTH is outlined in Algorithm 1.
**Algorithm 1.** The pseudo-code of the RTH.
1: ***Begin***
2: **Initialize:** the relevant parameters.
3: **Initialization:** random generation within the search space.
4:  While
$t<T_{max}$ do
5:    ***High-soaring stage:***
6:      Update the population by Equation (1)
7:    ***Low-soaring stage:***
8:      Update the population by Equation (5)
9:    ***Stooping and Swooping stage:***
10:      Update the population by Equation (8)
11:    t=t+1 12:  ***End while*** 13:  ***return*** best solution 14: ***end***

## 3. Proposed IRTH

The original RTH algorithm performs well in single-peaked functions and possesses a simple structure; however, it is prone to problems such as falling into local optimization when encountering complex real-world optimization problems. To overcome these problems, we propose an improved version of the RTH algorithm based on the trust domain. The details are as follows.

### 3.1. A Stochastic Reverse Learning Strategy Based on Bernoulli Mapping

In the RTH algorithm, the initial population is acquired by random initialization. The random initial population often leads to a more dispersed distribution of individuals in the solution space, which lacks a targeted exploration of the potential optimal solution region. Due to the randomness of the individuals in the random initial population, it is likely that most of the individuals gather in the region near the local optimal solution at the beginning, which makes the search less efficient. Therefore, in this subsection, we propose a stochastic backward learning strategy based on Bernoulli mapping to improve the RTH algorithm, which retains the original randomness while increasing the utilization of prior knowledge to enhance the performance of the algorithm.

**Figure 1:**
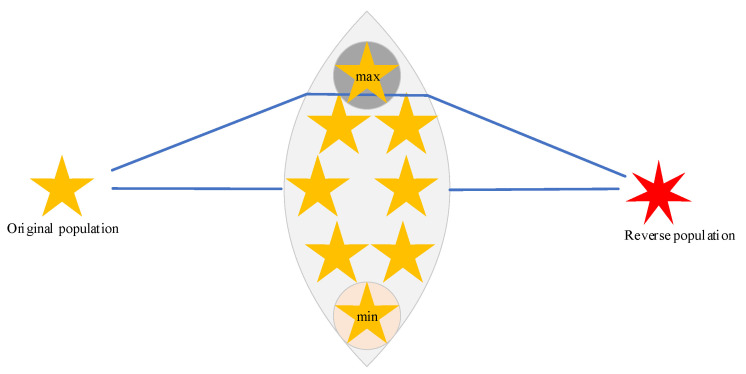
Graphical description of the stochastic reverse learning strategy.

Bernoulli transition mapping is a probabilistic transition mechanism that transforms the state of an individual based on the Bernoulli distribution. Mathematically, the Bernoulli distribution is a discrete probability distribution, and in this subsection, we improve it by using a two-part linear mapping, as shown in Equation (13).

(13) $$X_{n+1}=\{\begin{array}{c} \frac{x_{n}}{1-\sigma },\;\;0<x_{n}\leq 1-\sigma \\ \frac{x_{n}-\left(1-\sigma \right)}{\sigma },\;\;1-\sigma <x_{n}\leq 1 \end{array},$$

where σ is set to 0.4. Reverse learning is a strategy that enhances the search capability of an algorithm by considering the current solution and its inverse. In addition to evaluating and manipulating the regular solutions, the inverse solutions of these solutions are generated, and by comparing the fitness values of the original and the inverse solutions, the better solution is selected to proceed to the next round of iterations. If the fitness of the inverse solution is better than that of the original solution, then the original solution is replaced with the inverse solution, which allows the algorithm to potentially jump out of the local optimum since the inverse solution may be located in a more optimal region outside the current search region. The solution of the reverse solution is shown by Equation (14):

(14) $$OBL_{i}=K⊙\left(MAX+MIN\right)-X_{i},$$

where $OBL_{i}$ denotes the oppositional solution obtained by particle $X_{i}$ after refractive oppositional–mutual learning; K is a matrix of one row dim columns, where the elements in the matrix are random numbers between 0 and 1; and MAX and MIN are the maximum and minimum values of the individual, respectively. When the fitness value of the opposing solution is better than the original solution, the original solution is updated to the opposing solution; otherwise, no update is performed. This is shown in Equation (15).

(15) $$X_{i}=\{\begin{array}{c} OBL_{i},\;\;f\left(OBL_{i}\right)<f\left(X_{i}\right)\\ X_{i},\;\;other \end{array},$$

where $f\left(OBL_{i}\right)$ denotes the fitness value of the opposing solution and $f\left(X_{i}\right)$ denotes the fitness value of the original solution. Figure 1 graphically depicts this strategy for better understanding by the reader.

### 3.2. Dynamic Position Update Optimization Strategy for Stochastic Mean Fusion

**Figure 2:**
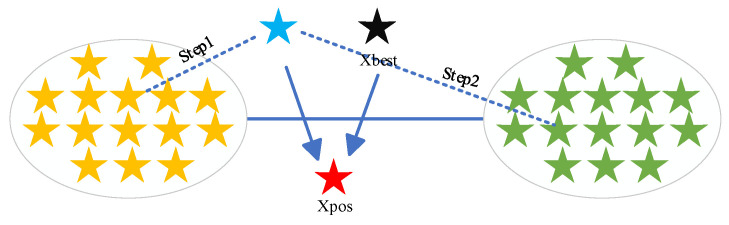
Dynamic position update optimization strategy diagram.

In the RTH algorithm, randperm is used to randomize the order of individuals in the population, and this randomness helps to introduce a certain amount of diversity, but also introduces uncertainty. Each time the algorithm is run, the difference in the randomized arrangement may lead to a large difference in the convergence path and final result of the algorithm. In addition to this, the fixed calculation of StepSize cannot be well adapted to different optimization problem scenarios. For some complex objective functions with different scale characteristics, a more flexible step-size adjustment strategy may be needed to ensure that the algorithm can converge globally faster. Therefore, we propose a dynamic position update optimization strategy for stochastic mean fusion, which ensures that the algorithm can better converge to the global optimal solution through two different step-size computation methods, and uses adaptive parameters to optimize the convergence speed of the algorithm in response to the dynamically changing environments and problems. The improved position update is shown in Equation (16).

(16) $$X\left(t\right)=\{\begin{array}{c} X_{best}+\alpha *step1.*R,\;\;rand>0.5\\ X_{best}+\alpha *step2.*R,\;\;other \end{array}$$

where R is randn(1,dim); α is an adaptive parameter, which is calculated by Equation (17); step1, step2 are two different dynamically varying step sizes to enable the algorithm to better adapt to different optimization problem scenarios, which are calculated by Equations (18) and (19).

(17) $$\alpha ={\left(1-\frac{t}{T_{max}}\right)}^{2*\frac{t}{T_{max}}},$$

(18) $$step1=X_{mean1}-X\left(t\right),$$

(19) $$step2=X_{mean2}-X\left(t\right)$$

where $X_{mean1}$ is obtained by taking a random number p between 5 and 10 and then randomly selecting p individuals in the population to form a new subpopulation $X_{p}$, and then finding the mean of $X_{p}$ as $X_{mean1}$. $X_{mean2}$ is obtained by taking a random number q between 15 and N and then randomly selecting q individuals in the population to form a new subpopulation $X_{q}$, and then finding the mean of $X_{q}$ as $X_{mean2}$. A schematic of the dynamic position update optimization strategy for stochastic mean fusion strategy is given in Figure 2.

### 3.3. Optimization Method for Frontier Position Update Based on Trust Domain

In the stooping and swooping stage, it is necessary for the algorithm to converge to the optimal solution as quickly as possible while also retaining some of the exploration to prevent the algorithm from falling into a local optimum. In the RTH algorithm, it is relatively complicated to use combinations of TF, G, etc. In single-peak function optimization problems, these complex combinations are too delicate to compute, leading to a waste of computational resources; in complex multi-peak function optimization problems, they may not be able to effectively adapt to the changes in the function terrain, making it difficult to accurately guide the search toward the global optimal solution.

The trust domain method can effectively prevent the algorithm divergence problem caused by too large a step size by approximating the objective function in a trust domain and finding the optimal solution in this region. As the algorithm iterates, the radius of the trust domain is dynamically adjusted according to the change in the fitness value to improve the global convergence performance of the algorithm. In addition, the adaptive adjustment of the radius can also adapt to different scenarios faster, so that the algorithm can perform well in all kinds of functions and problems. In the IRTH algorithm, the trust domain-based optimization method for the frontier position update is determined by Equation (20).

(20) $$X\left(t\right)=\{\begin{array}{c} X\left(t\right)+\left(X_{mean1}-TDX\left(R1\right)\right).*rand,\;\;rand>0.5\\ X\left(t\right)+\left(X_{mean2}-TDX\left(R1\right)\right).*rand,\;\;other \end{array}$$

where R1 is a random number that denotes a randomly selected individual in the trust domain for the position update of the auxiliary algorithm. TDX denotes the trust domain population, which contains each individual located in the trust domain. In each round of position update, we randomly select an individual in the trust domain for position update, which ensures the convergence speed of the algorithm while retaining the randomness, and well improves the performance of the algorithm.

**Figure 3:**
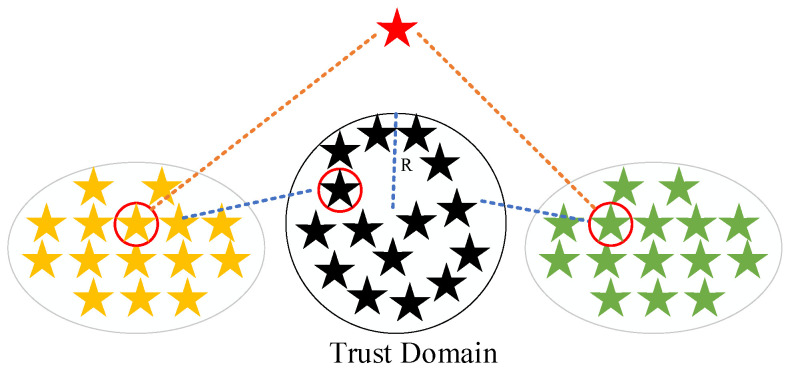
Optimization method for frontier position update based on trust domain diagram.

When the fitness value of our new solution is greater than the fitness value of the original solution, this indicates that there is a problem with the position update, and we are interested in increasing the radius of the trust domain to make the algorithm better to explore. When the fitness value of the new solution is less than the fitness value of the original solution, it means that the position update of the algorithm is effective, then we reduce the radius of the trust domain to make the algorithm converge faster. A schematic diagram of the trust domain-based optimization method for frontal position update is given in Figure 3.

**Figure 4:**
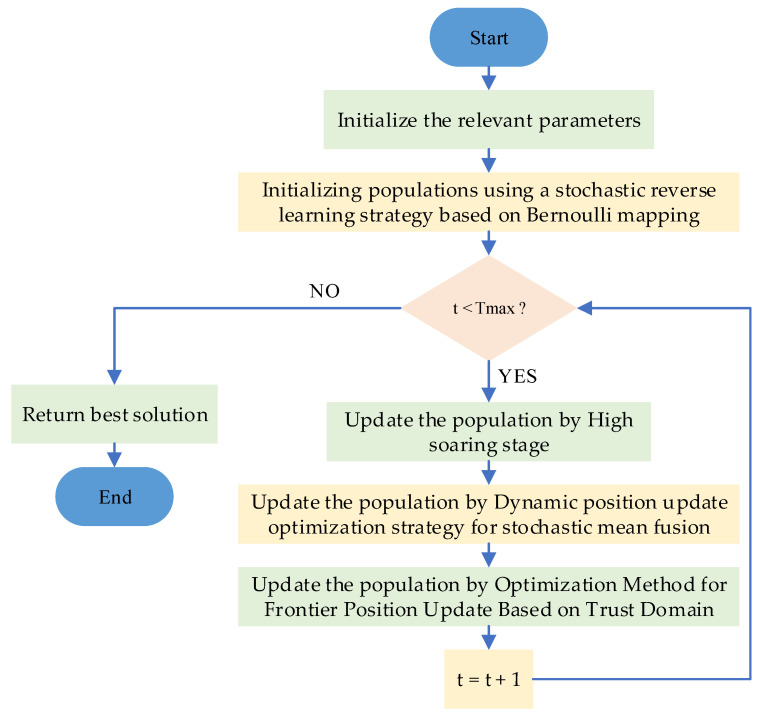
Flowchart of IRTH algorithm.

Figure 4 depicts the flowchart of the IRTH algorithm, while its pseudocode is provided in Algorithm 2.
**Algorithm 2.** The pseudo-code of the IRTH.
1: ***Begin***
2: **Initialize:** the relevant parameters.
3: **Initialization:** random generation within the search space.
4:   While $t<T_{max}$ do
5:    ***High-soaring stage:***
6:       Update the population by Equation (1)
6:    ***Dynamic position update optimization strategy for stochastic mean fusion***
6:      Update the population by Equation (16)
6:    ***Optimization Method for Frontier Position Update Based on Trust Domain***
8:      Update the population by Equation (20)
13:    t=t+1 14:  ***End while*** 15:  ***return*** best solution 16: ***end***

### 3.4. Computational Time Complexity

The performance of an algorithm is crucial, but it is equally important to assess its time complexity. In many optimization tasks, algorithms must not only deliver high performance but also demonstrate good real-time efficiency. Time complexity refers to how the runtime of an algorithm increases as the size of the input grows. Analyzing the time complexity of an optimization algorithm provides insight into the time overhead when handling large-scale problems. For the RTH algorithm, its time complexity mainly stems from the number of iterations and the stages of high soaring, low soaring, and stooping and swooping, each of which involves updating positions within the population. Therefore, the time complexity of RTH is O(T∗dim∗N). In the IRTH algorithm, since it only improves position updates without adding new factors that increase complexity, its time complexity remains O(T∗dim∗N).

## 4. Experimental Results and Detailed Analyses

In this section, we experimentally analyze the proposed IRTH algorithm using the CEC2017 test set. The benchmark function is very important for the performance evaluation of the algorithms, so we first introduce the CEC2017 test set [38]. Next, we present the parameter settings for each comparison algorithm. Then, we qualitatively analyze the IRTH algorithm. In addition, we conduct comparison experiments with 11 other algorithms using the CEC2017 test set. Finally, to fully validate the effectiveness of IRTH, we perform statistical analysis. To ensure the fairness and impartiality of the experiments, all the algorithm populations are set to 50, and the maximum number of iterations is set to 1000.

### 4.1. Benchmark Test Functions

The CEC2017 test set is widely used to evaluate the performance of optimization algorithms [39,40,41]. It covers a range of benchmark functions with different characteristics, including multimodal, unimodal, and high- and low-dimensional problems. It enables us to comprehensively evaluate the effectiveness of the algorithms on different types of problems.

Among them, two single-peak functions, seven simple multi-peak functions, ten hybrid functions, and ten composite functions are included, which are able to test the performance of the algorithms in different situations, providing a more comprehensive evaluation.

### 4.2. Competitor Algorithms and Parameter Settings

In this section, we evaluate the performance of the IRTH algorithm by comparing it with 11 advanced algorithms to demonstrate its strong capabilities. We have chosen two classical optimization algorithms, the chameleon swarm algorithm (CSA) and artificial gorilla troops optimizer (GTO); six novel optimization algorithms proposed in the last two years, including the secretary bird optimization algorithm (SBOA), snow ablation optimizer (SAO), rime optimization algorithm (RIME), gold rush optimizer (GRO), red-billed blue magpie optimizer (RBMO), and enterprise development-inspired metaheuristic (ED); and two improved versions of the optimization algorithm, the hyperheuristic whale optimization algorithm (HHWOA), improved grey wolf optimizer (IGWO), and the red-tailed hawk algorithm (RTH). A more comprehensive inclusion of existing optimization algorithms is provided by the selection of three types of algorithms. In the experiments, the superiority of the algorithms proposed in this paper can be comprehensively demonstrated.

In the comparison experiments, we set the parameter values of the algorithm according to reference. Table 2 summarizes the parameter settings of these algorithms for ease of reading and lists the references for all algorithm parameter settings for further review.

### 4.3. Qualitative Analysis of IRTH

In this subsection, we conduct a qualitative analysis of the proposed IRTH algorithm. Initially, we examine the diversity of the algorithm’s population, which plays a crucial role in exploring the unknown space effectively. Next, we evaluate the balance between exploration and exploitation, as the initial iterations require stronger exploration, while later iterations focus more on exploitation. We validate the performance of IRTH through experiments that measure both exploration and exploitation. Lastly, to assess the effectiveness of the improvements made, we perform ablation experiments. Detailed explanations are provided below.

#### 4.3.1. Analysis of the Population Diversity

In optimization algorithms, population diversity refers to the extent of variation among the individuals within a population [51]. These individuals usually represent possible solutions to the problem. If the diversity of the population is reduced, the algorithm may converge prematurely to a local optimum, limiting its ability to explore the global optimum. Conversely, maintaining a high level of population diversity allows the algorithm to search different regions of the solution space, enhancing the likelihood of finding the global optimum. In this subsection, we assess the population diversity of the IREH algorithm, which is calculated using Equation (21).

(21) $$I_{C}\left(t\right)=\sqrt{\sum_{i=1}^{N}\sum_{d=1}^{D}{\left(x_{id}\left(t\right)-c_{d}\left(t\right)\right)}^{2}},$$

where $I_{C}\left(t\right)$ denotes the population diversity, N represents the population size, D indicates the problem’s dimensionality, and $x_{id}\left(t\right)\;$ denotes the value of the i individual in the d dimension at the t iteration. $c_{d}\left(t\right)$ reflects the degree of dispersion of the entire population relative to the center of mass at the t iteration. $c_{d}\left(t\right)\;$is calculated through Equation (22).

(22) $$c_{d}\left(t\right)=\frac{1}{D}\overset{N}{\underset{i=1}{\sum }}x_{id}\left(t\right).$$

**Figure 5:**
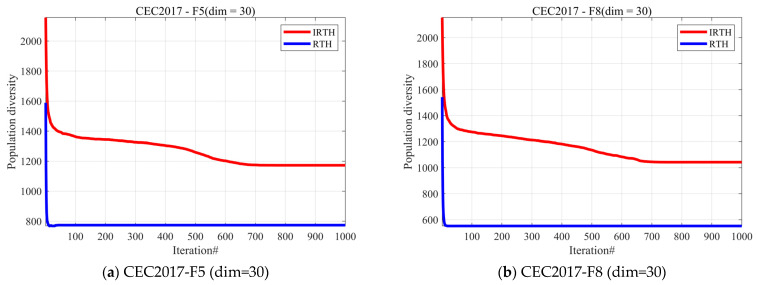

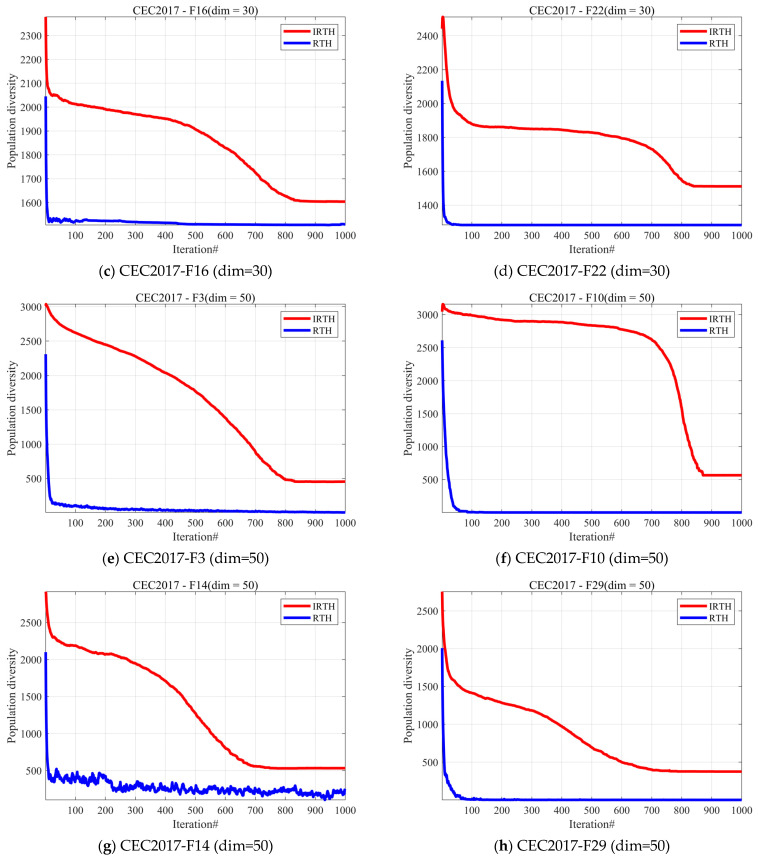

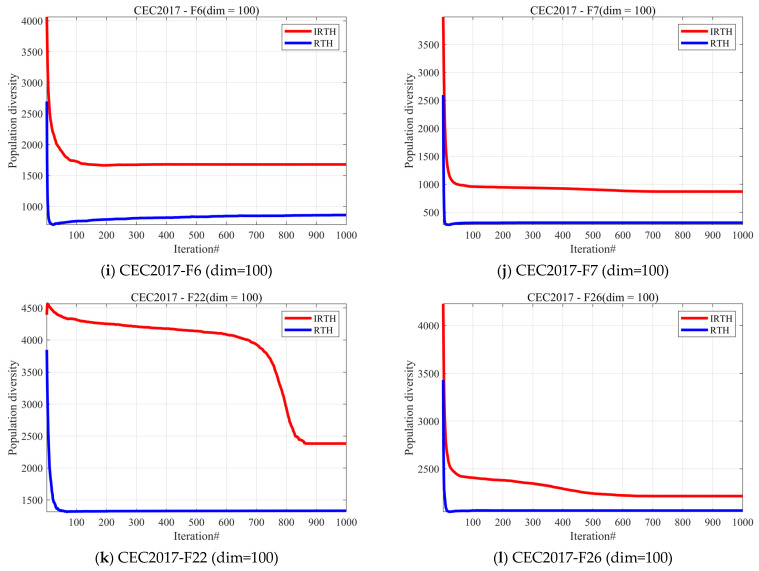
The analysis of the population diversity of IRTH and RTH.

Figure 5 shows the experimental results of population diversity analysis for both algorithms, from which it can be seen that the population diversity of the IRTH algorithm is due to the RTH algorithm in most cases. Compared to the RTH algorithm, whose population diversity mostly decreases to a very low value within 100 generations, the IRTH algorithm’s population diversity decreases slower and is able to maintain the diversity of the population well.

#### 4.3.2. Analysis of the Exploration and Exploitation

In optimization algorithms, both exploration and exploitation play crucial roles. Exploration involves the algorithm performing a broad search of the solution space, aiming to uncover diverse regions, including unknown areas that may contain globally optimal solutions. Exploitation, on the other hand, focuses on conducting a localized search around the best solutions discovered, refining them further. It leverages existing knowledge to delve deeper into regions deemed promising. If the algorithm over-explores, it may waste time searching the entire solution space aimlessly, missing opportunities to find better solutions in specific areas. Conversely, excessive exploitation can cause the algorithm to prematurely converge to a local optimum, preventing the discovery of potentially better solutions elsewhere in the solution space [52]. Thus, balancing exploration and exploitation is crucial for achieving optimal performance in the algorithm. In this subsection, we examine the exploration and exploitation aspects of the IRTH algorithm. Equations (23) and (24) calculate the percentage of exploration and exploitation.

(23) $$Exploration\left(\%\right)=\frac{Div\left(t\right)}{Div_{max}}\times 100\%,$$

(24) $$Exploitation\left(\%\right)=\frac{\left|Div\left(t\right)-Div_{max}\right|}{Div_{max}}\times 100\%,$$

where Div(t) denotes the measure of diversity at the tth iteration, which is calculated by Equation (25), and $Div_{max}$ denotes the maximum measure of diversity throughout the iteration.

(25) $$Div\left(t\right)=\frac{1}{D}\sum_{d=1}^{D}\frac{1}{N}\sum_{i=1}^{N}|median\left(x_{d}\left(t\right)\right)-x_{id}\left(t\right)|.$$

**Figure 6:**
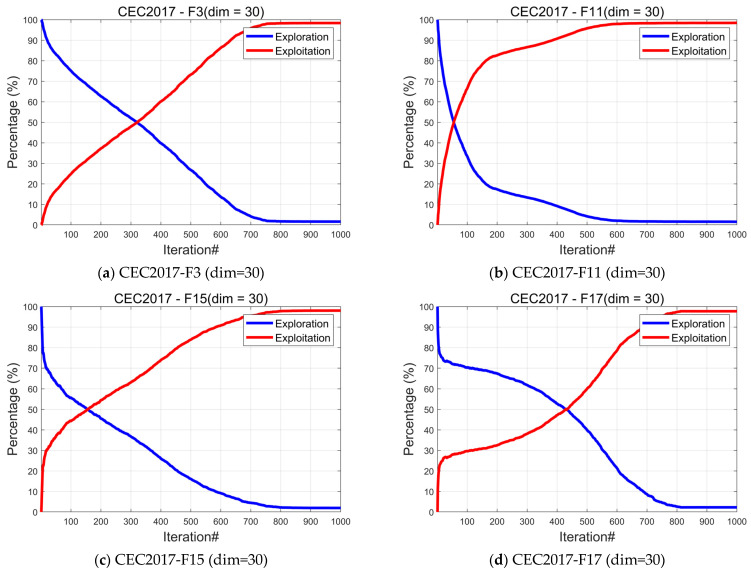

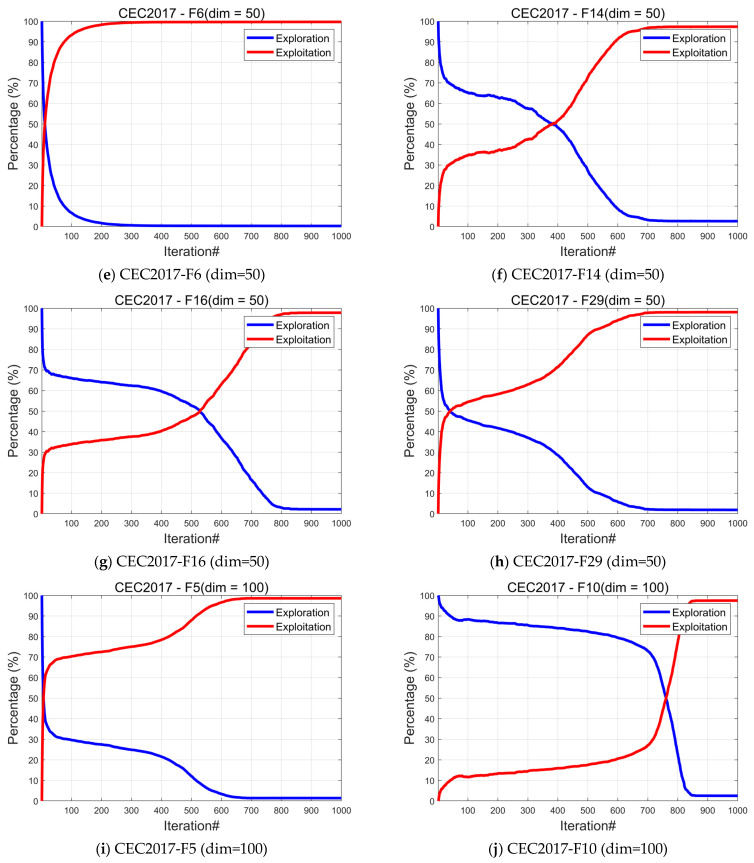

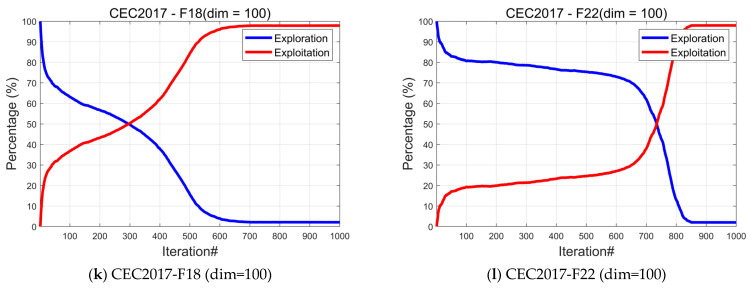
The analysis of the exploration and exploitation of IRTH.

The experimental results are presented in Figure 6. During the early stages of algorithm iteration, the proportion of exploration is significantly higher than that of exploitation. However, as the algorithm progresses, the proportion of exploitation gradually increases while the proportion of exploration decreases. By the end of the iteration, the proportion of exploitation approaches 100%, indicating that IRTH effectively balances exploration and exploitation, demonstrating strong performance in both aspects.

#### 4.3.3. Impact Analysis of the Modification

**Figure 7:**
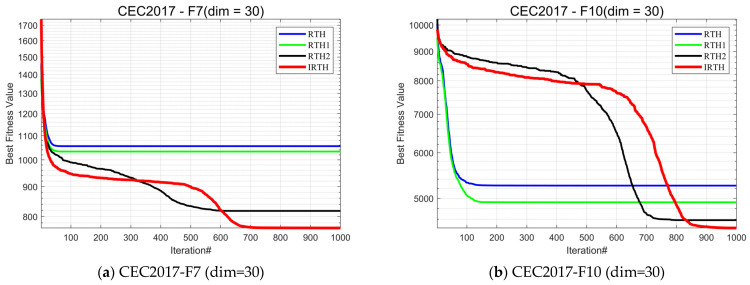

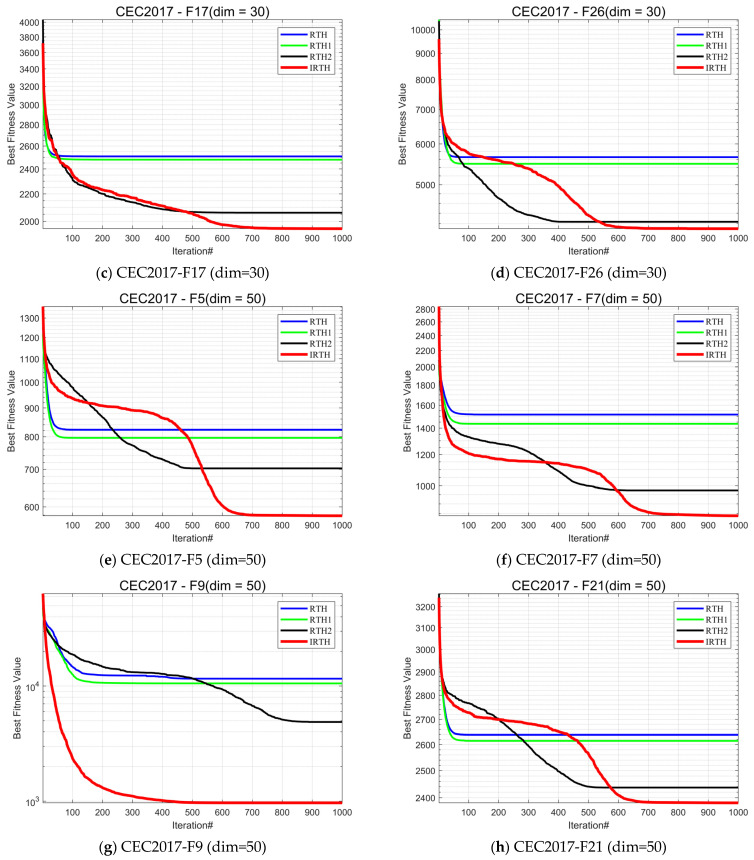

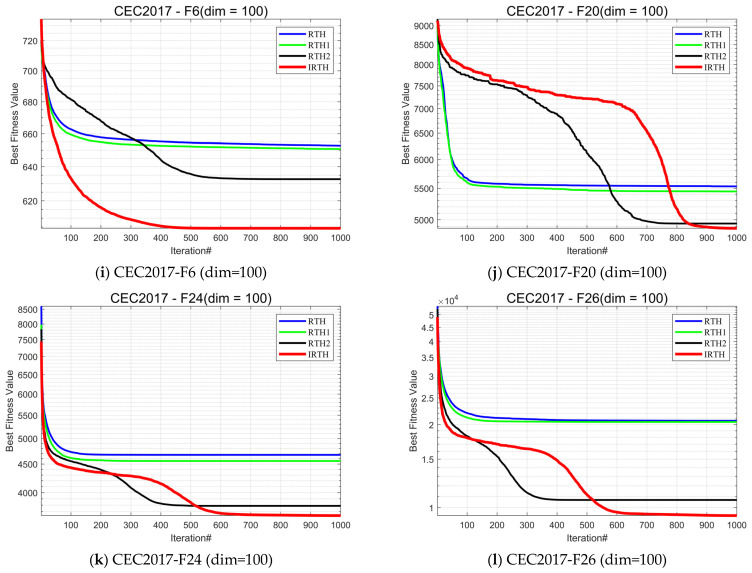
Comparison of different improvement strategies.

To assess the effectiveness of the strategy introduced in this paper, in this subsection, we conduct ablation experiments. When new strategies, operations, or parameters are introduced into an existing optimization algorithm, ablation experiments can be used to verify whether these additions are really effective. It enables us to objectively evaluate the value of new strategies. In this section, the algorithm with the addition of a stochastic reverse learning strategy based on a Bernoulli mapping strategy is named RTH1, the algorithm with the addition of a dynamic position update optimization strategy for stochastic mean fusion is named RTH2, and the algorithm that combines the three strategies with RTH is named IRTH. The experimental results are shown in Figure 7.

As can be seen from the figure, although IRTH does not obtain the fastest convergence speed for some functions, its convergence accuracy is the highest. In particular, on functions such as F6 and F9, IRTH improves its convergence accuracy along with its convergence speed, gaining a significant victory compared to the RTH algorithm. In addition to this, it is also evident from the experimental results that all three improvement strategies we proposed have good results against the RTH algorithm, with RTH1 outperforming RTH, RTH2 outperforming RTH1, and IRTH obtaining the best results in most of the functions. It can be proved that all three strategies we proposed are effective, and better results can be obtained by combining them.

### 4.4. Comparison Using CEC 2017 Test Functions

**Figure 8:**
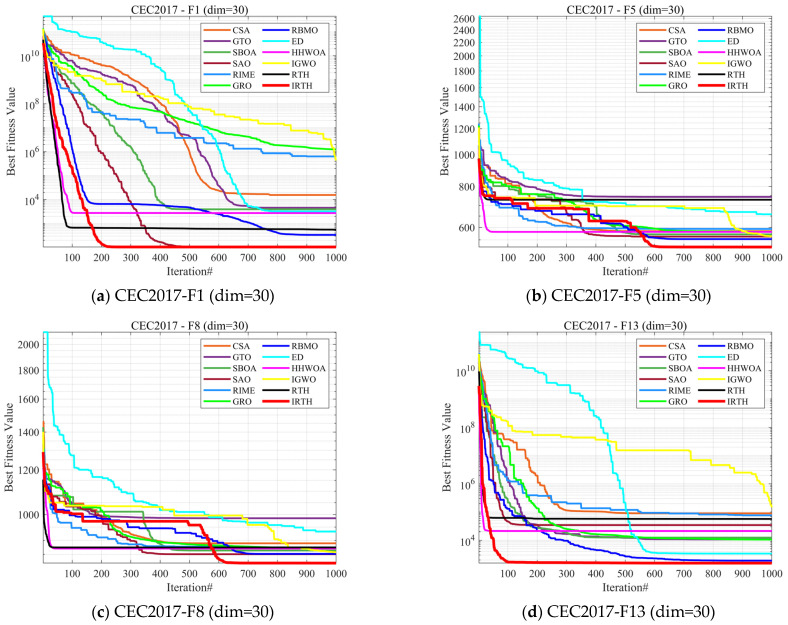

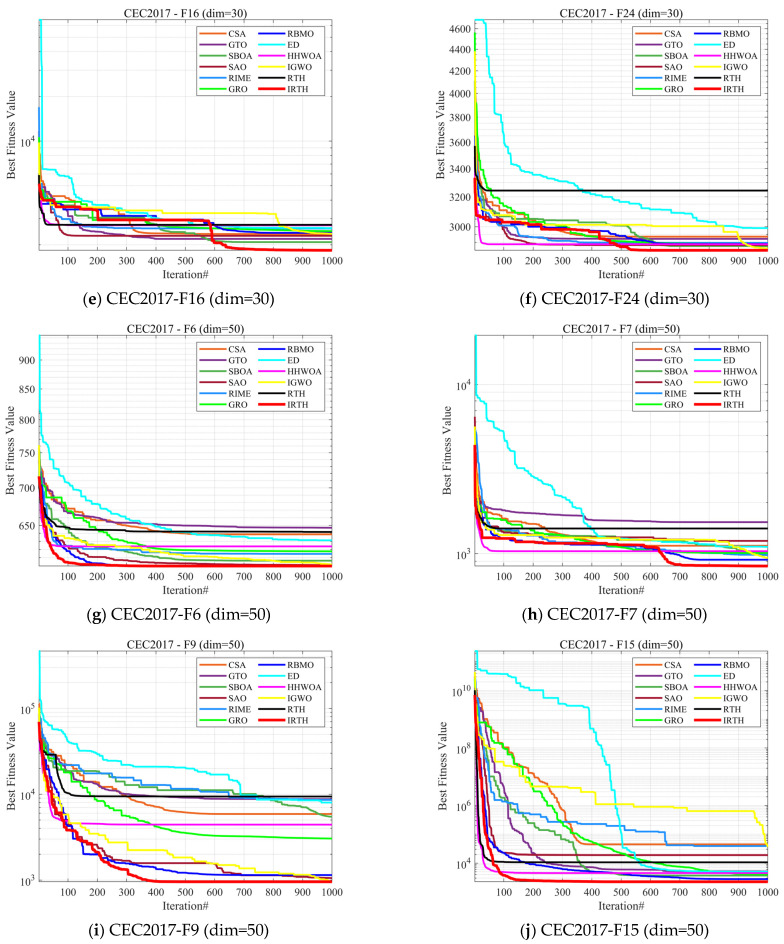

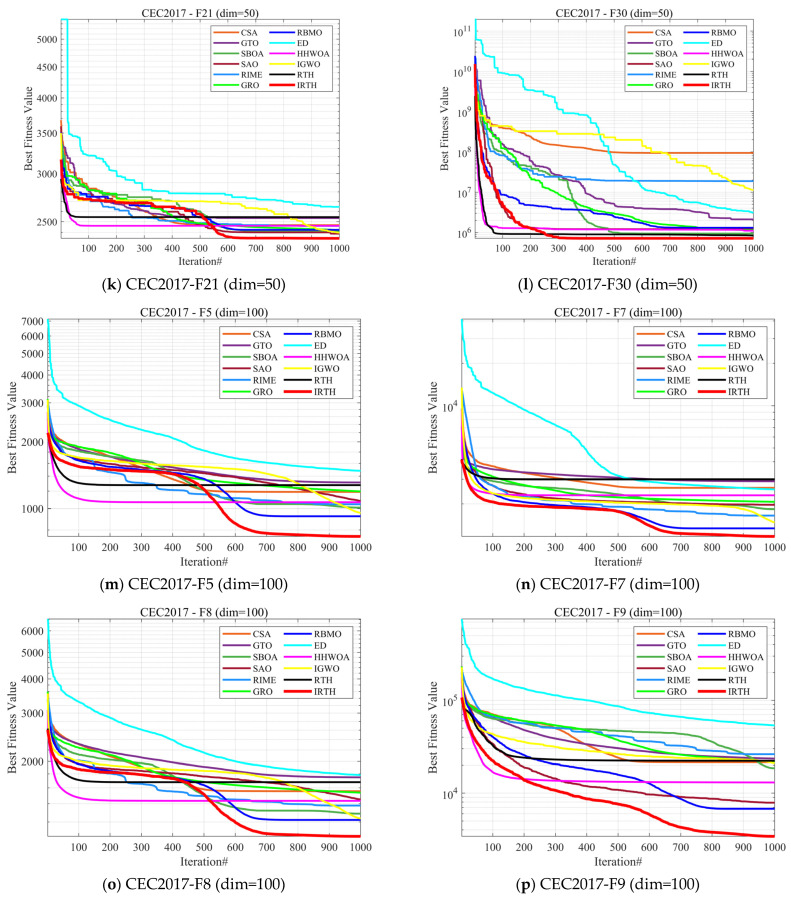

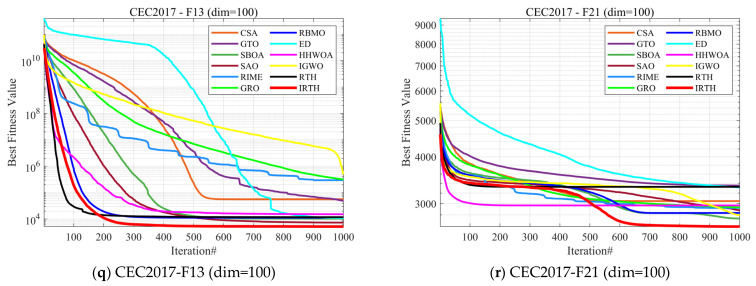
Comparison of convergence speed of different algorithms on CEC2017 test set.

**Figure 9:**
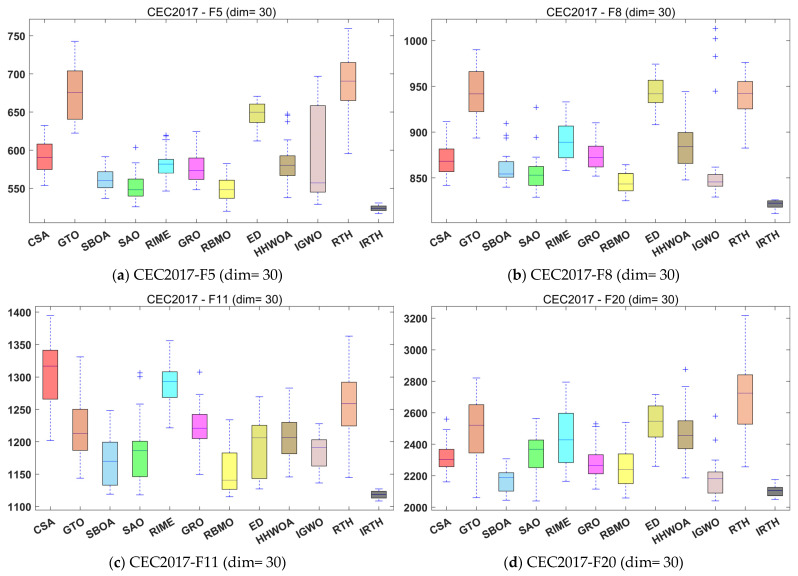

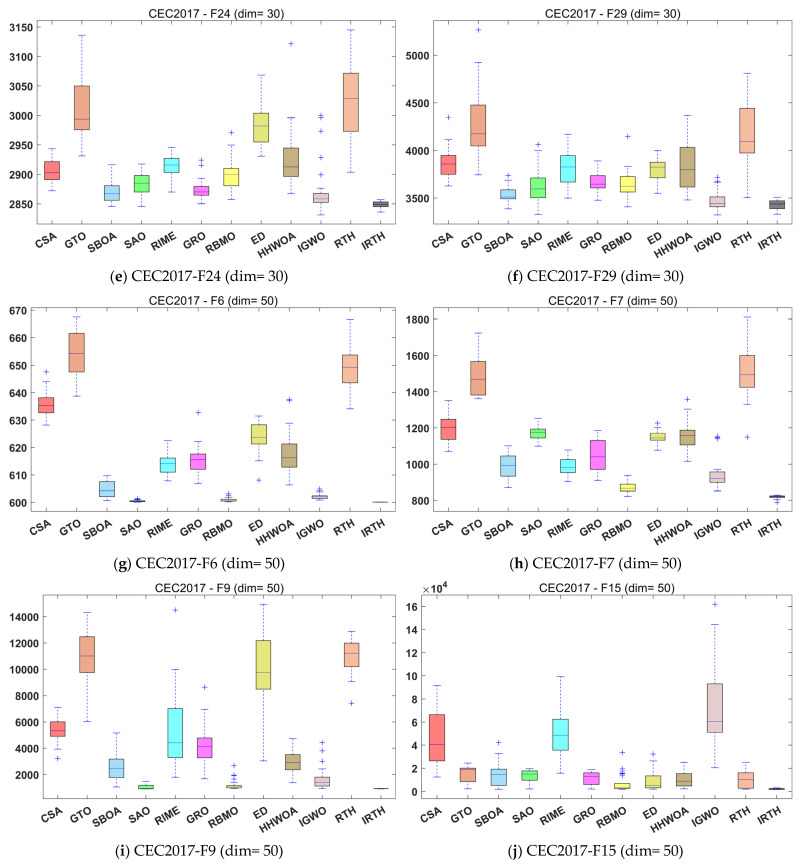

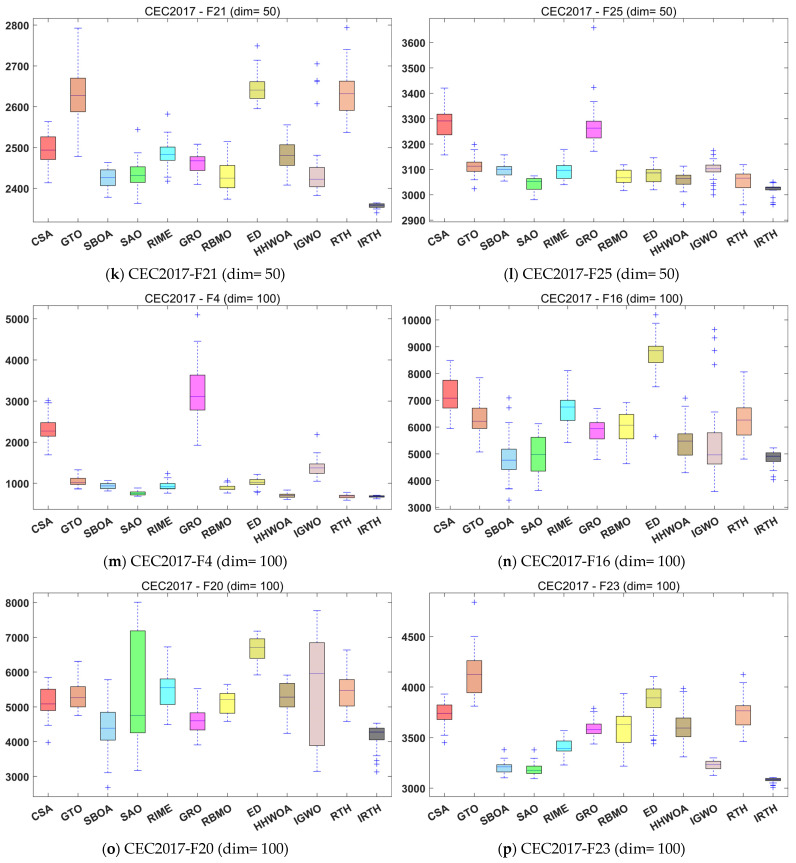

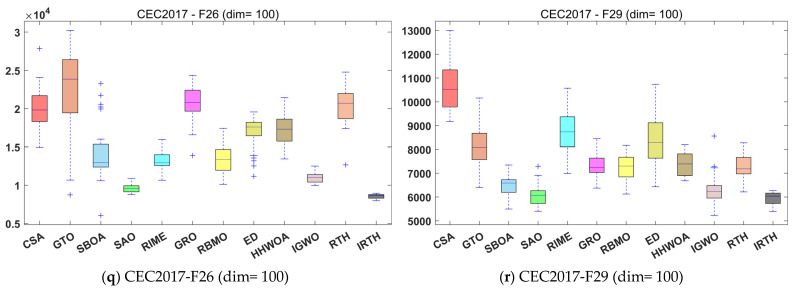
Boxplot analysis for different algorithms on the CEC2017 test set.

In this subsection, we validate the effectiveness of the algorithm by conducting experiments using three dimensions from the CEC2017 test set. We compare the proposed IRTH algorithm with 11 other state-of-the-art algorithms. These comparison experiments clearly highlight the strengths and weaknesses of IRTH, with the experimental numerical results presented in Table 3, Table 4 and Table 5. To visualize the convergence speed of the algorithms during the optimization process, the convergence graphs of all 12 algorithms are displayed in Figure 8. To minimize the impact of randomness and further assess the stability of the algorithms, the boxplot diagrams for all the algorithms are shown in Figure 9.

As shown in Figure 8, in the 30-dimensional case, although the convergence accuracy of IRTH is similar to that of SAO for the F7 function, the IRTH algorithm improves convergence speed by about 200 iterations. Similarly, for the F13 and F24 functions, the convergence accuracy of IRTH is comparable to that of some other comparative algorithms, but its convergence speed is much faster. Furthermore, for functions such as F5, F8, and F16, the convergence accuracies of IRTH are significantly superior to those of other comparative algorithms. In the 50-dimensional case, on the F6, F9, F15, and F30 functions, the algorithm’s convergence speed is greatly improved, and it is able to escape local optima towards the end, finding better solutions on both the F7 and F30 functions. Especially when compared to the RTH algorithm, IRTH shows significant improvements in both convergence speed and accuracy. Most importantly, the IRTH algorithm performs exceptionally well in higher dimensions, and in the 100-dimensional case, IRTH significantly outperforms other comparative algorithms in both convergence accuracy and speed.

From Figure 9, it can be seen that IRTH has good stability whether it is in 30, 50, or 100 dimensions. The mean, median, maximum, and minimum values of the 30 runs outperform the other compared algorithms to a great extent, obtaining competitive results.

### 4.5. Statistical Analysis

Statistical analysis is essential for optimizing algorithms, allowing researchers to assess and compare the effectiveness of different algorithms, which helps in choosing the most appropriate one for a given research problem. In this section, we apply the Wilcoxon rank sum test and the Friedman mean rank test to evaluate the performance of the IRTH algorithm, as detailed below.

#### 4.5.1. Wilcoxon Rank Sum Test

In this subsection, we employ the Wilcoxon rank sum test [53] to evaluate the IRTH algorithm to identify significant differences between it and other algorithms, without the assumption of a normal distribution. Unlike the traditional t-test, the Wilcoxon rank sum test is more flexible as it does not require normally distributed data, making it particularly useful for datasets with outliers or non-normal distributions. The Wilcoxon rank sum test statistic W is calculated by Equation (26).

(26) $$W=\overset{n_{1}}{\underset{i=1}{\sum }}R\left(X_{i}\right),$$

where $R\left(X_{i}\right)$ denotes the rank of $X_{i}$ among all observations. The test statistic U is calculated by Equation (27).

(27) $$U=W-\frac{n_{1}\left(n_{1}+1\right)}{2}.$$

For larger sample sizes, U is approximately normally distributed by Equations (28) and (29).

(28) $$\mu_{U}=\frac{n_{1}n_{2}}{2},$$

(29) $$\sigma_{U}=\sqrt{\frac{n_{1}n_{2}\left(n_{1}+n_{2}+1\right)}{12}},$$

and the standardized statistic Z is calculated by Equation (30).

(30) $$Z=\frac{U-\mu_{U}}{\sigma_{U}}.$$

We set the significance level at 0.05, assessing whether the results from each IRTH run significantly differ from those of other algorithms at this level. The null hypothesis ($H_{0}$) assumes no significant difference between the two algorithms. If p<0.05, we reject the null hypothesis, indicating a significant difference; otherwise, we accept the null hypothesis, suggesting no significant difference. The experimental results are presented in Table 6, where IRTH shows significant advantages. “+” means IRTH is superior to the comparison algorithm in the Wilcoxon rank sum test, “=” means the two algorithms are not much different, and “−” means IRTH is inferior to the comparison algorithm in the Wilcoxon rank sum test.

#### 4.5.2. Friedman Mean Rank Test

In this subsection, we apply the Friedman mean rank test [54] to assess the ranking of IRTH. This nonparametric method is commonly used to compare the median differences across three or more related samples. The friedman mean rank test is especially in repeated measures designs or paired samples and is often preferred over ANOVA when the data do not follow a normal distribution.

The Friedman nonparametric criterion statistic is defined by Equation (31).

(31) $$Q=\frac{12}{nk\left(k+1\right)}\overset{k}{\underset{j=1}{\sum }}R_{j}^{2}-3n\left(k+1\right),$$

where n is the number of blocks, k is the number of groups, and $R_{j}$ is the rank sum for j-th group. When n and k are large, Q approximately follows a $\chi^{2}$ distribution with k−1 degrees of freedom.

**Figure 10:**
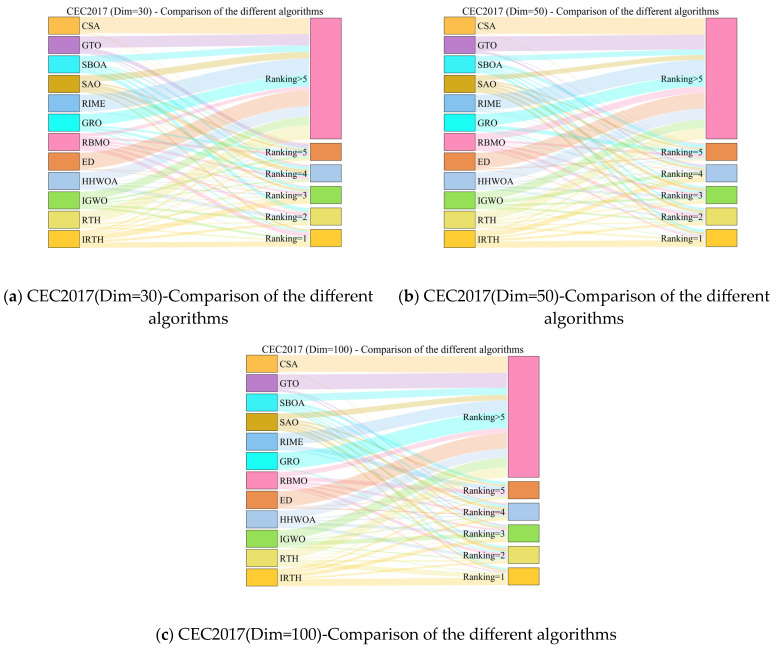
The ranking Sankey of different algorithms on CEC2017.

The experimental results are provided in Table 7, with the ranking distribution depicted in Figure 10. M.R represents the average ranking of the algorithm across 30 functions, and T.R denotes the final overall ranking. From the experimental results, it is evident that the IRTH algorithm performs excellently, achieving the top overall ranking in 30-, 50-, and 100-dimensional cases.

### 4.6. Sensitivity Analysis of Parameters

**Figure 11:**
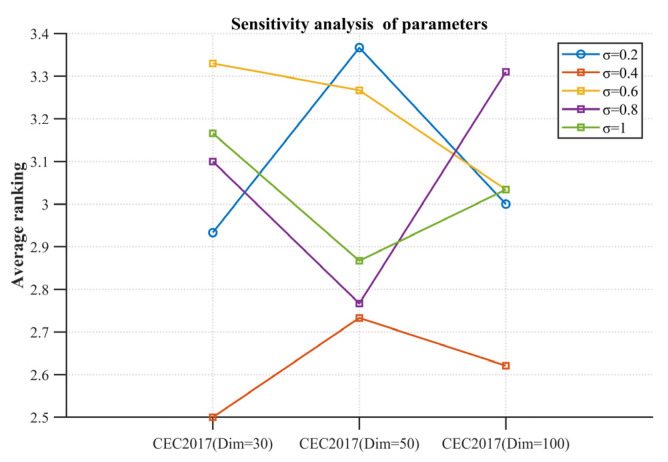
Parametric sensitivity analysis average ranking graph.

In this section, we present an analysis of the parameter σ. Experiments were conducted using 30, 50, and 100 dimensions of the CEC2017 test set, with experimental parameters consistent with those used previously. We calculated the average ranking of different values of σ for each dimension and illustrated it as a curve in Figure 11. From the experiment results, it is evident that the optimal overall effect occurs when σ=0.4. Therefore, in this paper, we set s to 0.4.

## 5. IRTH Algorithm for Real-Environment UAV Path Planning

In this section, in order to verify the effectiveness of the IRTH algorithm in planning the path of UAVs in real environments, we use the IRTH algorithm to solve the problem of planning the path of a UAV in a real environment. Firstly, we model the real environment, and secondly, we use the algorithm to solve the problem. The specific details are as follows.

### 5.1. Scenarios and Objective Functions

In this subsection, we present the scenarios and objective functions for the UAV flight environment.

#### 5.1.1. Scenario Setting

**Figure 12:**
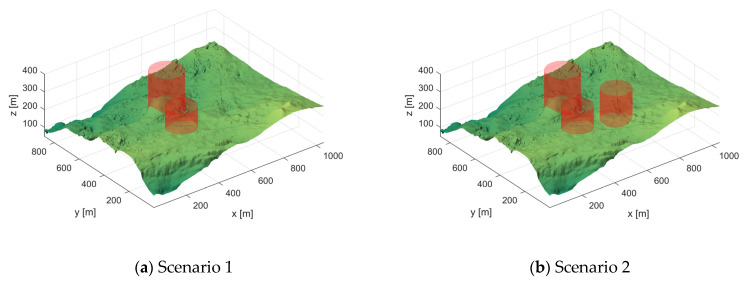

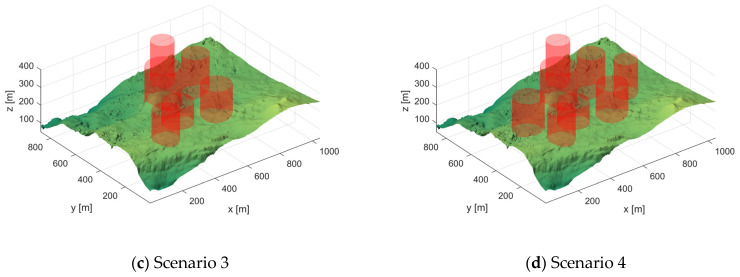
Four different scenario views.

In this section, the scenarios we use to evaluate the performance of the algorithms are derived from real digital elevation model maps from LiDAR sensors. Two regions with different terrain structures on Christmas Island, Australia, were selected and then augmented to generate four baseline scenes, as shown in Figure 12. The red cylinders indicate the number and location of threats.

#### 5.1.2. Optimization Problem Definition

In this subsection, we define the UAV path-planning problem as a cost function which includes path length, safety and feasibility constraints, etc., as detailed below.

1. Path Length Costs

For UAV path planning, the shortest path is a very important metric, but in most real-world problems, there are often many obstacles in the straight line from the start point to the end point, so we need to perform obstacle-avoidance path planning for UAVs. In this subsection, we assume that the flight waypoint of the UAV is $P_{ij}=\left(x_{ij},y_{ij},z_{ij}\right)$. The Euclidean distance between two waypoints is $\vec{∥P_{ij}P_{i,j+1}∥}$. Therefore, the flight cost of the UAV is given by Equation (32).

(32) $$F_{1}\left(X_{i}\right)=\overset{n-1}{\underset{j=1}{\sum }}∥\vec{P_{i,j}P_{i,j+1}}∥,$$

2. Threat Costs

In the UAV path-planning problem, threat cost is one of the important factors affecting the decision. In various complex environments, UAVs usually face a variety of potential threats, and the introduction of threat cost can enable planning algorithms to avoid high-threat areas during path selection, reduce the risk of being attacked or damaged, and improve the survivability of UAV missions. Therefore, the threat cost of the UAV is calculated by Equation (33).

(33) $$F_{2}\left(X_{i}\right)=\overset{n-1}{\underset{j=1}{\sum }}\overset{K}{\underset{k=1}{\sum }}T_{k}\left(\vec{P_{ij}P_{i,j+1}}\right),$$

where $T_{k}\left(p_{ij}p_{i,j+1}\right)$ denotes flight constraint costs, which are calculated by Equation (34).

(34) $$T_{k}\left(p_{ij}p_{i,j+1}\right)=\{\begin{array}{c} 0,\;\;d_{k}>S+D+R_{k}\\ \left(S+D+R_{k}\right)-d_{k},\;\;D+R_{k}<d_{k}\leq S+D+R_{k}\\ \infty ,\;\;d_{k}\leq D+R_{k} \end{array},$$

where $R_{k}$ denotes the radius of the Kth cylindrical obstacle, D denotes the peripheral collision region, and $d_{k}$ denotes the distance from the center of the obstacle to the path $L_{p_{ij}p_{i+1,j}}$.

3. High Costs

In UAV path planning, altitude cost is an important factor affecting path selection. UAV flight altitude can directly affect the strength and coverage of communication signals. Lower altitudes may be interfered with by terrain or obstacles, affecting communication stability and mission control effectiveness. Introducing altitude cost can enable the planning algorithm to better select the flight altitude for communication stability and ensure the reliability of mission data transmission. The altitude cost of the UAV is calculated by Equation (35).

(35) $$F_{3}\left(X_{i}\right)=\overset{n}{\underset{j=1}{\sum }}H_{ij},$$

where $H_{ij}$ denotes the cost of the height of the $X_{i}$ location, which is calculated by Equation (36).

(36) $$H_{ij}=\{\begin{array}{cc} \left|h_{ij}-\frac{\left(h_{max}+h_{min}\right)}{2}\right|,\; & \;h_{min}\leq h_{ij}\leq h_{max}\\ \infty ,\; & \;\mathrm{otherwise} \end{array},$$

where $h_{ij}$ is the altitude at which the UAV is located; $h_{min}$ is the minimum altitude at which flight is allowed; and $h_{max}$ is the maximum altitude at which flight is allowed.

4. Smoothness Costs

In UAV path planning, the smoothness cost is an important metric used to measure the smoothness or continuity of path turns. UAVs are subject to inertial and dynamical constraints in flight, with limited changes in turning radius or speed. The smoothness cost ensures the enforceability of path planning by avoiding sharp turns that do not meet the physical constraints of the UAV at the path planning stage. The smoothing cost for UAV flight is calculated by Equation (37).

(37) $$F_{4}\left(X_{i}\right)=a_{1}\overset{n-2}{\underset{j=1}{\sum }}\alpha_{ij}+a_{2}\overset{n-1}{\underset{j=1}{\sum }}\left|\beta_{ij}-\beta_{i,j-1}\right|,$$

where $a_{1}$ denotes the UAV horizontal turn angle constraint penalty coefficient, and $a_{2}$ denotes the UAV vertical pitch angle constraint penalty coefficient. $\alpha_{ij}$ denotes the horizontal turn angle constraint, which is computed by Equation (38). $\beta_{ij}$ denotes the vertical pitch angle, which is computed by Equation (39).

(38) $$\alpha_{ij}=\arctan\frac{L_{p_{ij}^{\prime }p_{i,j+1}^{\prime }}\times L_{p_{ij}^{\prime }p_{i,j+2}^{\prime }}}{L_{p_{ij}^{\prime }p_{i,j+1}^{\prime }}\cdot L_{p_{ij}^{\prime }p_{i,j+2}^{\prime }}},$$

(39) $$\beta_{ij}=\arctan\frac{Z_{i,j+1}-Z_{ij}}{∥L_{p_{ij}p_{i,j+1}}∥},$$

where $L_{p_{ij}^{\prime }p_{i,j+1}^{\prime }}$ is their projections on the plane, which is calculated by Equation (40).

(40) $$L_{p_{ij}^{\prime }p_{i,j+1}^{\prime }}=k\times \left(L_{p_{ij}p_{i,j+1}}\times k\right),$$

where k is the unit vector in the positive direction of the axis.

5. Overall Objective Function (OEF)

Considering the path length cost, threat cost, high cost, and smoothness cost, the overall objective function based on multi-cost is calculated by Equation (41).

(41) $$F\left(X_{i}\right)=\overset{4}{\underset{k=1}{\sum }}b_{k}*F_{k}\left(X_{i}\right).$$

where $b_{k}$ is the weight coefficient.

6. Problem Formulation

Based on the four aforementioned cost and overall objective functions, the goal of this system is to minimize the cost of flying the UAV. Therefore, the optimization problem is formulated by Equation (42).

(42) $$P:\min F\left(X_{i}\right)=\overset{4}{\underset{k=1}{\sum }}b_{k}*F_{k}\left(X_{i}\right).$$

(42a) $$s.t.\overset{4}{\underset{k=1}{\sum }}b_{k}.$$

In our experiments, in the UAV path-planning problem, the path cost is the single most important factor that best characterizes the quality of the planned path. Next is the threat cost; only good avoidance of threatening factors can make our UAVs perform their tasks well. So, we define the weight coefficients as 0.4, 0.3, 0.2, and 0.1.

#### 5.1.3. Analysis of Experimental Results

In order to verify the performance of IRTH, we conducted experiments on it in four different scenarios. The specific details are as follows.

**Figure 13:**
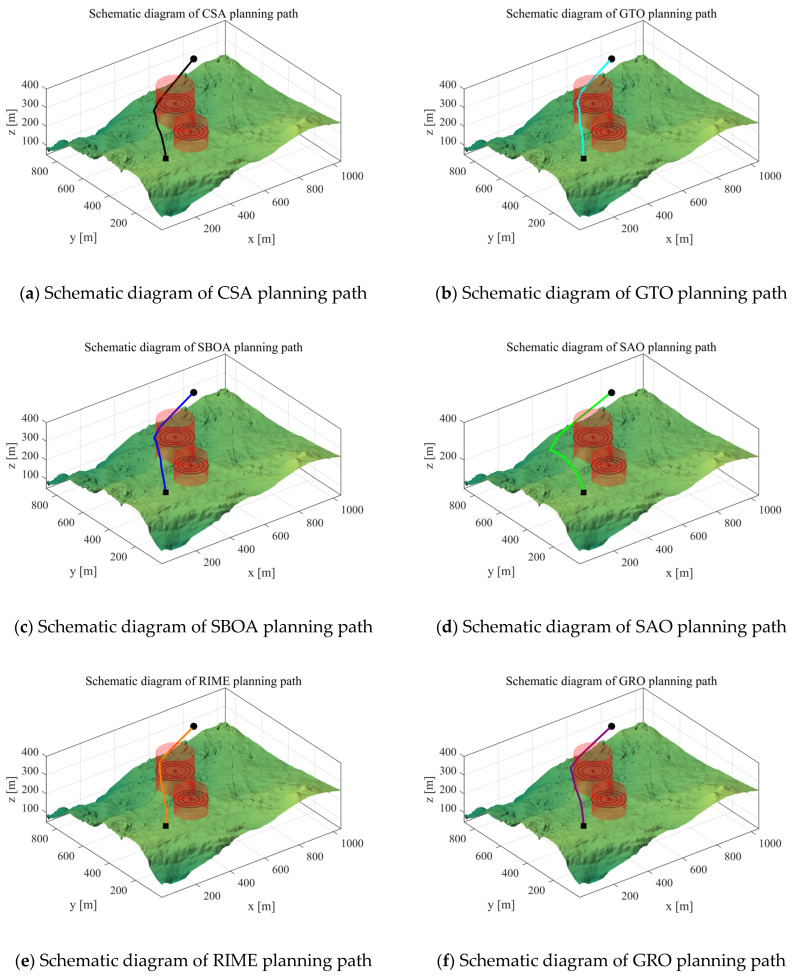

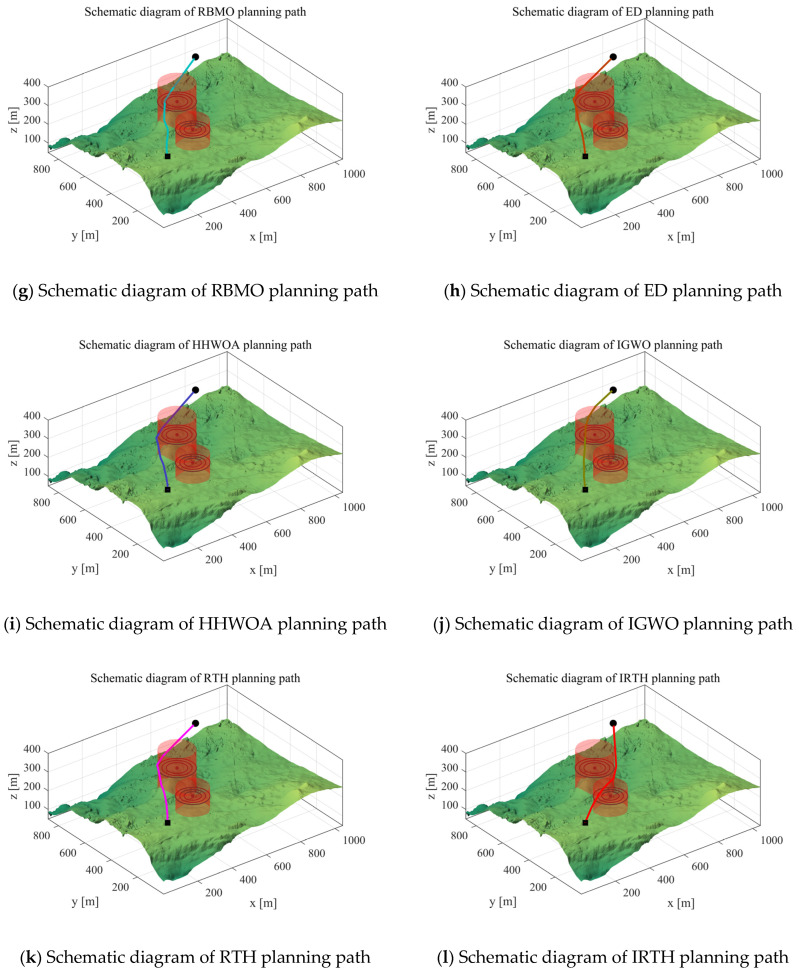
Scenario 1: Schematic diagram of path planning.

Scenario 1: In this subsection, we perform an experimental analysis to verify the performance of the algorithm in the case of Scenario 1. We set the starting point of the UAV to [100,100,150], the end point to [800,800,150], and the waypoints to 10 for experimental analysis. Its path cost is shown in Table 8, where mean, median, max, and min denote the mean, median, maximum, and minimum values obtained from 30 independent runs of the algorithm, respectively. The path-planning schematic for each algorithm in Scenario 1 is shown in Figure 13.

From the experimental results, it can be seen that the IRTH algorithm obtains good performance in Scenario 1. The total cost of IRTH is 414.26, which is the lowest among the 12 algorithms in terms of the mean value of 30 runs. In terms of stability, the difference between the minimum and maximum values of the IRTH algorithm is 5.59, which is also the most stable and can be good for path planning for the UAV in Scenario 1.

**Figure 14:**
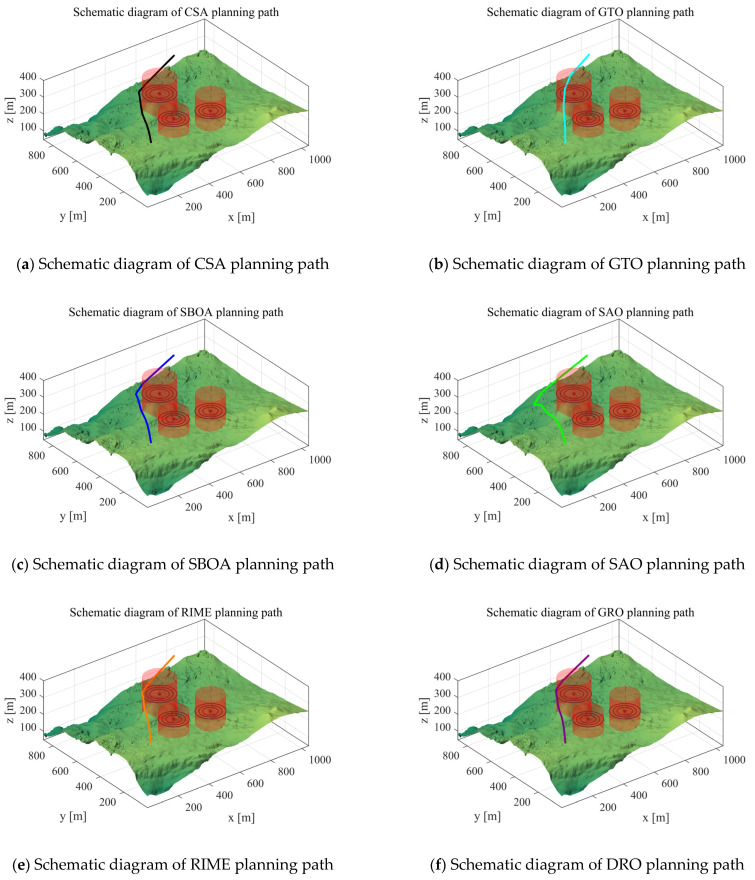

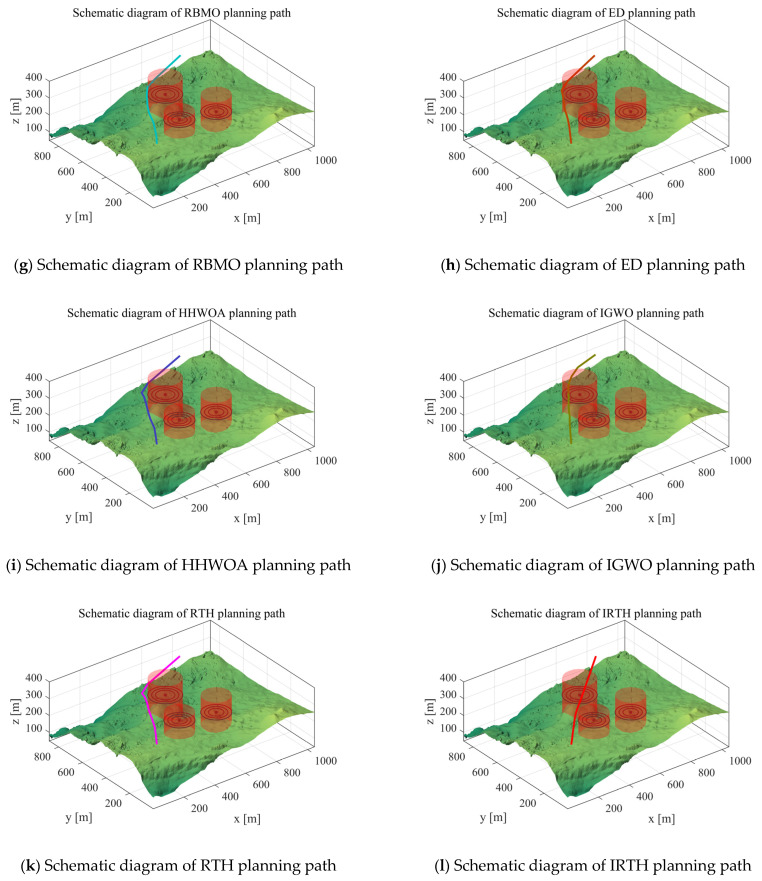
Scenario 2: Schematic diagram of path planning.

Scenario 2: In this subsection, we perform an experimental analysis to verify the performance of the algorithm in the case of Scenario 2. We set the starting point of the UAV to [100,100,150], the end point to [800,800,150], and the waypoints to 10 for experimental analysis. Its path cost is shown in Table 9. The path-planning schematic for each algorithm in Scenario 2 is shown in Figure 14.

As can be seen from the experimental results, the IRTH still obtains competitive results in Scenario 2. Although the SBOA, SAO, ED, and HHWOA algorithms are more stable compared to TRTH, IRTH obtains the best results in terms of mean value. The mean value has a cost improvement of 30 over IGWO, proving its superior performance.

**Figure 15:**
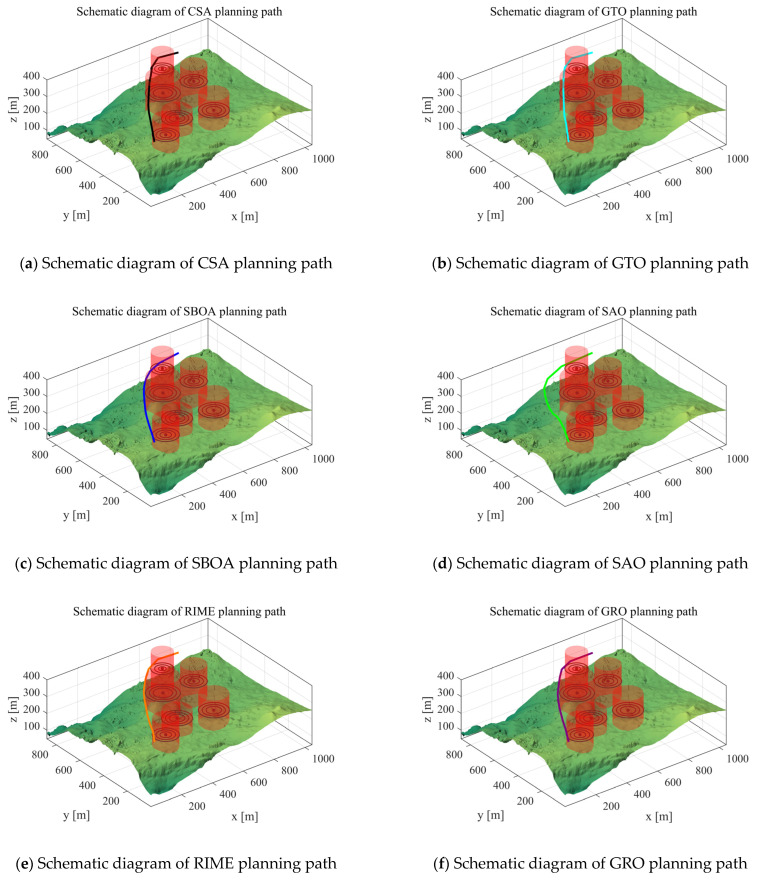

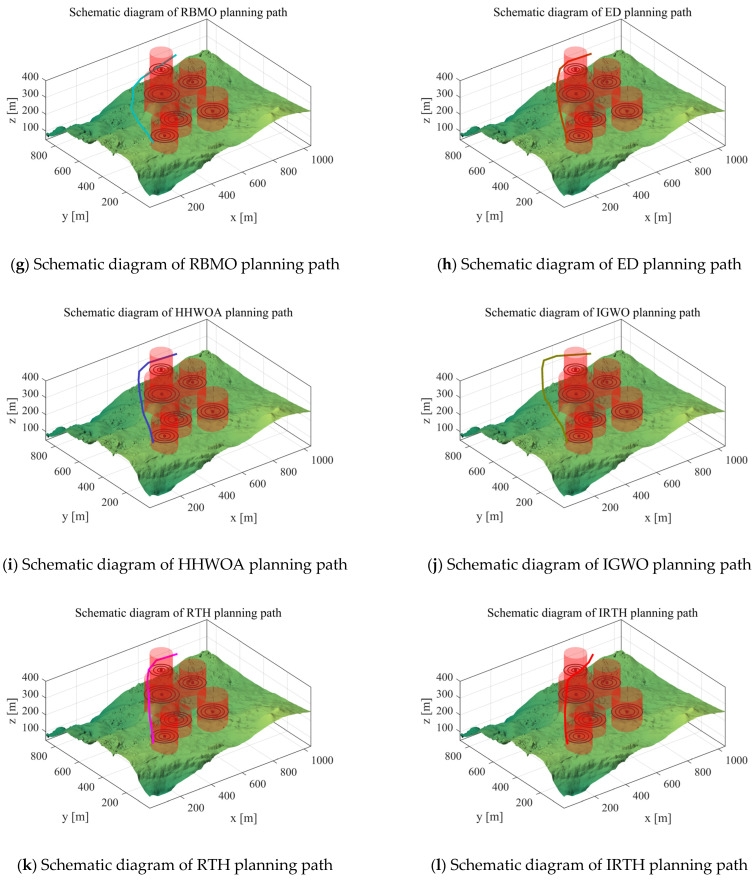
Scenario 3: Schematic diagram of path planning.

Scenario 3: In this subsection, we perform an experimental analysis to verify the performance of the algorithm in the case of Scenario 3. We set the starting point of the UAV to [100,100,150], the end point to [800,800,150], and the waypoints to 10 for experimental analysis. Its path cost is shown in Table 10. The path-planning schematic for each algorithm in Scenario 3 is shown in Figure 15.

From the experimental results, it can be seen that IRTH still obtains competitive results in Scenario 3. Except for the IRTH algorithm, the cost of the paths planned by the rest of the algorithms is greater than 430, while the IRTH algorithm plans a path with a cost of 426.47. Most importantly, the RBMO and IGWO algorithms are not able to plan paths for the UAV in some cases, which proves the wide applicability of the IRTH.

**Figure 16:**
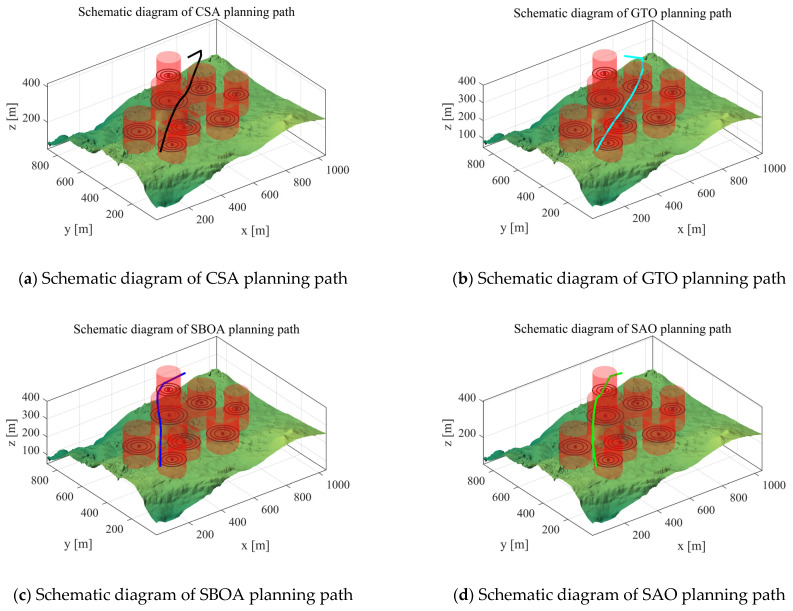

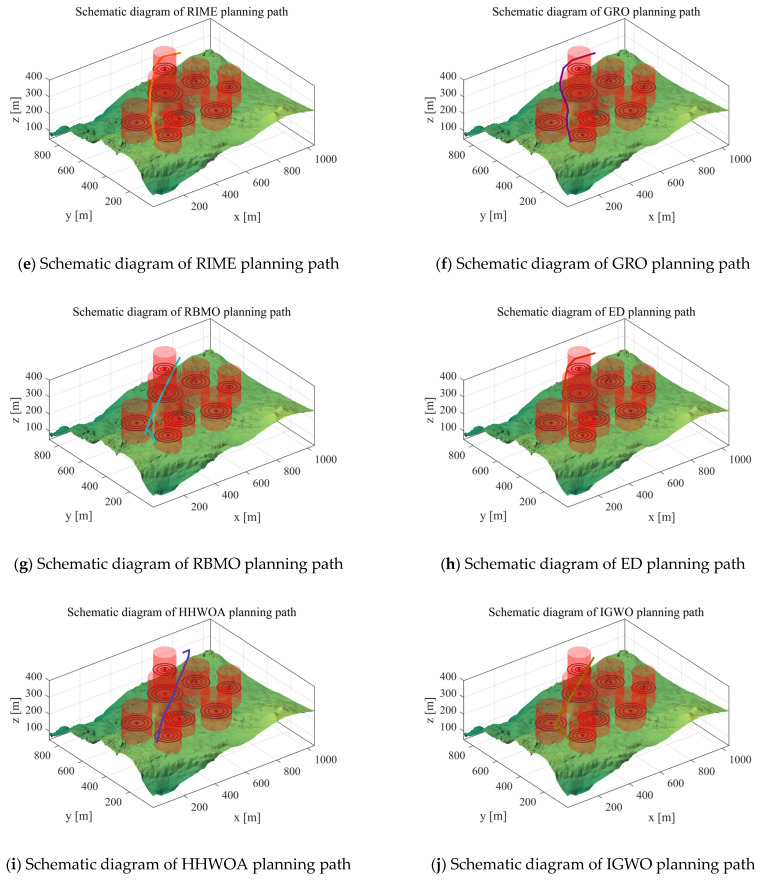

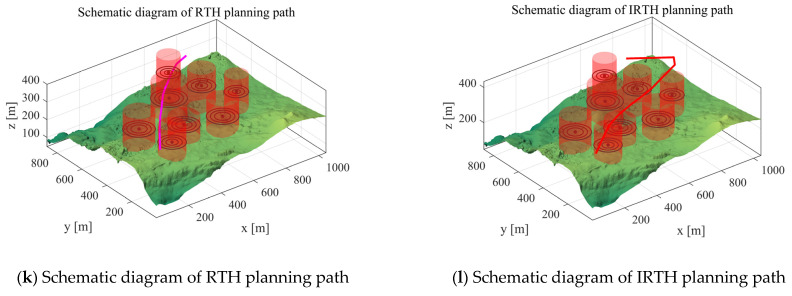
Scenario 4: Schematic diagram of path planning.

Scenario 4: In this subsection, we perform an experimental analysis to verify the performance of the algorithm in the case of Scenario 4. We set the starting point of the UAV to [100,100,150], the end point to [800,800,150], and the waypoints to 10 for experimental analysis. Its path cost is shown in Table 11. The path-planning schematic for each algorithm in Scenario 4 is shown in Figure 16.

From the experimental results, it can be seen that IRTH still obtains competitive results in Scenario 4. From Scenario 1 and Scenario 2, it seems that all algorithms are able to perform path planning for the UAV, but as the problem becomes more complex and the number of obstacles increases, the two algorithms in Scenario 3 and four algorithms in Scenario 4 are not able to perform path planning. However, IRTH is still able to perform path planning for the UAV and obtains the best results by planning the path with the lowest cost among the 12 compared algorithms.

## 6. Conclusions

In this paper, an improved RTH algorithm based on a trust domain is proposed for the UAV path-planning problem in real environments. Firstly, we adopt the Bernoulli mapping-based backward learning strategy, a dynamic position update optimization strategy for stochastic mean fusion, and an optimization method for frontier position update based on the trust domain, three strategies to improve the performance of the algorithm in terms of population diversity, convergence speed, and convergence accuracy, so that the algorithm can solve the problem better. In addition, to verify the effectiveness of the algorithms, the performance of the algorithms is comprehensively evaluated using the CEC2017 test set. Finally, the algorithm is applied to the UAV path-planning problem in a real environment to perform path planning for UAVs.

In the future, based on the excellent performance of the IRTH algorithm, it is planned to apply it to other practical engineering problems to solve problems that need to be solved in other fields—for example, cloud resource scheduling problems, neural network feature selection problems, and so on.

## Figures and Tables

**Table 1:** Summary of the latest optimization algorithms.

Algorithm	Inspired	Classification	Reference
Alpha evolution (AE)	The alpha operator combines an adaptive base vector with step sizes that are both random and adaptive.	Evolutionary	[22]
Exponential-Trigonometric Optimization (ETO)	An intricate blend of exponential and trigonometric functions.	Mathematical-based	[23]
Hunger Games Search (HGS)	The hunger-induced actions and behavioral decisions of animals.	Swarm-based	[24]
weIghted meaN oF vectOrs (INFO)	The weighted mean idea.	Mathematical-based	[25]
Parrot Optimizer (PO)	Notable behaviors exhibited by trained Pyrrhura molinae parrots.	Swarm-based	[26]
Polar Lights Optimization (PLO)	Based on the Northern Lights, this paper introduces Polar Lights Optimization (PLO) for solving optimization problems.	Physics-based	[17]
Information acquisition optimizer (IAO)	Human information acquisition behaviors involve three key strategies.	Swarm-based	[27]
Weighted average algorithm (WAA)	The weighted average position for the whole population.	Mathematical-based	[28]
The Neural Population Dynamics Optimization Algorithm (NPDOA)	Brain neuroscience.	Swarm-based	[15]
Four-Vector Intelligent Metaheuristic (FVIM)	Four top-performing leaders within a swarm.	Swarm-based	[29]
Flood algorithm (FLA)	The complex dynamics and flow patterns of water masses during river basin floods.	Swarm-based	[30]
Frilled Lizard Optimization (FLO)	The distinctive hunting strategies of frilled lizards in their native environment.	Swarm-based	[31]
Arctic puffin optimization (APO)	The flight patterns and underwater foraging habits of Arctic puffins.	Swarm-based	[32]
Eel and Grouper Optimizer (EGO)	The cooperative interaction and foraging tactics of eels and groupers in marine ecosystems.	Swarm-based	[33]
Pied kingfisher optimizer (PKO)	The unique hunting strategies and symbiotic relationships exhibited by pied kingfishers in their natural environment.	Swarm-based	[34]

**Table 2:** Parameter settings of the comparison algorithms.

Algorithms	Parameter Name	Parameter Value	Reference
CSA	v,rho,gamma, alpha	0.1,1.0,2.0,4.0	[42]
GTO	P , Beta, w	0.03,3,0.8	[43]
SBOA	beta	1.5	[44]
SAO	k	1	[45]
RIME	W	5	[46]
GRO	sigma_initial	2	[47]
RBMO	Epsilon	0.5	[48]
ED	g	1	[16]
HHWOA	w	3	[49]
RTH	A , R0 , r	15,0.5,1.5	[50]

**Table 3:** Results of various algorithms tested on the CEC 2017 benchmark (dim = 30).

ID	Metric	CSA	GTO	SBOA	SAO	RIME	GRO	RBMO	ED	HHWOA	IGWO	RTH	IRTH
F1	mean	2.5268× 10^4^	5.0944× 10^3^	5.6114× 10^3^	4.0570× 10^3^	3.9372× 10^5^	2.3385× 10^6^	7.0808× 10^2^	2.9724× 10^3^	2.5445× 10^3^	4.3980× 10^5^	5.3680× 10^3^	1.7175× 10^3^
	std	3.1327× 10^4^	5.8841× 10^3^	5.9302× 10^3^	4.0759× 10^3^	2.2904× 10^5^	5.2160× 10^6^	7.8384× 10^2^	2.4910× 10^3^	3.5655× 10^3^	2.7221× 10^5^	5.0480× 10^3^	1.7021× 10^3^
F3	mean	3.2621× 10^4^	1.0753× 10^3^	6.7930× 10^3^	6.6223× 10^4^	5.6882× 10^3^	3.4128× 10^4^	3.0090× 10^2^	8.0829× 10^4^	3.0000× 10^2^	5.1794× 10^3^	3.0000× 10^4^	8.3308× 10^2^
	std	8.4141× 10^3^	9.5157× 10^2^	4.1221× 10^3^	1.7565× 10^4^	3.3352× 10^3^	8.4822× 10^3^	7.8950× 10^-1^	1.5840× 10^4^	1.4593× 10^-2^	3.3919× 10^3^	2.0762× 10^-9^	2.6554× 10^4^
F4	mean	5.2383× 10^2^	4.8913× 10^2^	4.9312× 10^2^	4.9602× 10^2^	5.0150× 10^2^	5.1239× 10^2^	4.8577× 10^2^	4.9204× 10^2^	4.7482× 10^2^	4.9921× 10^2^	4.2103× 10^2^	4.7185× 10^2^
	std	3.4100× 10^1^	2.6032× 10^1^	2.4609× 10^1^	1.6715× 10^1^	2.0474× 10^1^	1.7495× 10^1^	1.4386× 10^1^	2.2995× 10^1^	3.2282× 10^1^	1.3031× 10^1^	2.9404× 10^1^	2.1995× 10^1^
F5	mean	5.9570× 10^2^	6.8035× 10^2^	5.5860× 10^2^	5.5188× 10^2^	5.7958× 10^2^	5.7873× 10^2^	5.5124× 10^2^	6.4815× 10^2^	5.8568× 10^2^	5.6833× 10^2^	6.6768× 10^2^	5.3147× 10^2^
	std	1.6985× 10^1^	3.8921× 10^1^	1.2993× 10^1^	1.3567× 10^1^	1.9803× 10^1^	1.8263× 10^1^	1.4611× 10^1^	1.9400× 10^1^	2.0295× 10^1^	4.8565× 10^1^	3.4121× 10^1^	6.1281× 10^0^
F6	mean	6.1964× 10^2^	6.4306× 10^2^	6.0034× 10^2^	6.0002× 10^2^	6.0449× 10^2^	6.0393× 10^2^	6.0009× 10^2^	6.0343× 10^2^	6.0522× 10^2^	6.0047× 10^2^	6.4057× 10^2^	6.0000× 10^2^
	std	4.4496× 10^0^	8.2688× 10^0^	5.2448× 10^-1^	5.5258× 10^-2^	2.5034× 10^0^	1.4079× 10^0^	2.3822× 10^-1^	3.5219× 10^0^	3.6660× 10^0^	2.8919× 10^-1^	7.4497× 10^0^	1.8704× 10^-3^
F7	mean	8.9373× 10^2^	1.0750× 10^3^	8.0717× 10^2^	8.3401× 10^2^	8.2481× 10^2^	8.1598× 10^2^	7.8010× 10^2^	8.7468× 10^2^	8.6456× 10^2^	8.2044× 10^2^	1.0637× 10^3^	7.5878× 10^2^
	std	3.3413× 10^1^	7.7119× 10^1^	3.2540× 10^1^	6.8141× 10^1^	2.1257× 10^1^	3.3944× 10^1^	1.2747× 10^1^	1.7801× 10^1^	3.8633× 10^1^	5.6422× 10^1^	6.8721× 10^1^	4.7457× 10^0^
F8	mean	8.7336× 10^2^	9.4986× 10^2^	8.5854× 10^2^	8.6081× 10^2^	8.8801× 10^2^	8.6351× 10^2^	8.4982× 10^2^	9.4873× 10^2^	8.8213× 10^2^	8.8031× 10^2^	9.3960× 10^2^	8.3472× 10^2^
	std	1.1732× 10^1^	2.7647× 10^1^	1.7663× 10^1^	1.5274× 10^1^	2.1151× 10^1^	1.4949× 10^1^	1.4148× 10^1^	1.4678× 10^1^	2.0213× 10^1^	5.5719× 10^1^	2.6524× 10^1^	8.2683× 10^0^
F9	mean	1.7543× 10^3^	3.6873× 10^3^	9.6105× 10^2^	9.1016× 10^2^	1.7802× 10^3^	1.2072× 10^3^	9.0813× 10^2^	1.2338× 10^3^	1.2185× 10^3^	9.1110× 10^2^	4.2867× 10^3^	9.0145× 10^2^
	std	3.5944× 10^2^	8.5773× 10^2^	9.0969× 10^1^	2.8312× 10^1^	1.1670× 10^3^	3.1820× 10^2^	7.0088× 10^0^	2.4491× 10^2^	2.4972× 10^2^	2.2092× 10^1^	6.8867× 10^2^	8.2351× 10^-1^
F10	mean	4.9250× 10^3^	5.6615× 10^3^	4.0576× 10^3^	3.7100× 10^3^	4.4685× 10^3^	4.1210× 10^3^	4.6323× 10^3^	5.0919× 10^3^	4.7873× 10^3^	6.4184× 10^3^	5.1349× 10^3^	4.4389× 10^3^
	std	6.9564× 10^2^	1.0034× 10^3^	6.5293× 10^2^	5.8129× 10^2^	5.4907× 10^2^	5.9292× 10^2^	6.2115× 10^2^	3.1000× 10^2^	7.0310× 10^2^	2.2205× 10^3^	6.6262× 10^2^	5.1252× 10^2^
F11	mean	1.2986× 10^3^	1.2297× 10^3^	1.1767× 10^3^	1.1661× 10^3^	1.2948× 10^3^	1.2046× 10^3^	1.1664× 10^3^	1.1865× 10^3^	1.2019× 10^3^	1.1795× 10^3^	1.2611× 10^3^	1.1477× 10^3^
	std	5.3558× 10^1^	4.7065× 10^1^	3.7780× 10^1^	4.1435× 10^1^	6.0997× 10^1^	3.6968× 10^1^	3.1578× 10^1^	3.9749× 10^1^	4.9937× 10^1^	2.7819× 10^1^	6.0999× 10^1^	2.3393× 10^1^
F12	mean	1.6852× 10^7^	1.5626× 10^5^	5.9525× 10^5^	3.6164× 10^5^	1.0177× 10^7^	1.0509× 10^6^	1.8974× 10^4^	3.3351× 10^5^	4.3021× 10^4^	1.8439× 10^6^	2.3416× 10^4^	4.5370× 10^4^
	std	1.8270× 10^7^	1.2979× 10^5^	5.1766× 10^5^	3.2484× 10^5^	5.5728× 10^6^	7.9535× 10^5^	1.6458× 10^4^	2.9926× 10^5^	2.3693× 10^4^	1.5455× 10^6^	1.4730× 10^4^	1.7524× 10^4^
F13	mean	9.6833× 10^4^	2.0404× 10^4^	1.9129× 10^4^	2.1458× 10^4^	5.1582× 10^4^	2.1763× 10^4^	6.3328× 10^3^	3.5871× 10^4^	1.7913× 10^4^	1.1293× 10^5^	1.8770× 10^4^	5.8237× 10^3^
	std	4.7553× 10^4^	2.3427× 10^4^	1.9776× 10^4^	1.9010× 10^4^	4.3704× 10^4^	1.5582× 10^4^	1.3187× 10^4^	2.1632× 10^4^	1.8586× 10^4^	6.5731× 10^4^	2.1165× 10^4^	5.1114× 10^3^
F14	mean	2.4833× 10^3^	2.7080× 10^3^	9.5137× 10^3^	2.8358× 10^4^	3.4860× 10^4^	2.0049× 10^4^	1.4566× 10^3^	3.2602× 10^4^	1.4693× 10^3^	8.3857× 10^3^	1.7526× 10^3^	5.0657× 10^3^
	std	1.6214× 10^3^	1.9598× 10^3^	9.1744× 10^3^	2.5111× 10^4^	3.0256× 10^4^	2.0971× 10^4^	1.0199× 10^1^	2.1784× 10^4^	3.0598× 10^1^	6.5497× 10^3^	1.5094× 10^2^	2.6795× 10^3^
F15	mean	1.8524× 10^4^	6.5373× 10^3^	1.1582× 10^4^	5.4142× 10^3^	1.5255× 10^4^	5.7455× 10^3^	1.6705× 10^3^	3.4160× 10^3^	1.5549× 10^3^	1.9376× 10^4^	8.2909× 10^3^	6.0515× 10^3^
	std	8.4935× 10^3^	8.7413× 10^3^	1.1323× 10^4^	4.7554× 10^3^	1.1794× 10^4^	4.6040× 10^3^	7.0039× 10^1^	3.2952× 10^3^	6.8061× 10^1^	1.2344× 10^4^	9.7063× 10^3^	4.3110× 10^3^
F16	mean	2.4702× 10^3^	2.6175× 10^3^	2.1408× 10^3^	2.4225× 10^3^	2.5512× 10^3^	2.1658× 10^3^	2.2663× 10^3^	2.9281× 10^3^	2.4505× 10^3^	2.0412× 10^3^	2.8202× 10^3^	2.1409× 10^3^
	std	2.5701× 10^2^	3.1808× 10^2^	3.5859× 10^2^	3.1980× 10^2^	2.8263× 10^2^	2.5067× 10^2^	2.8021× 10^2^	1.3966× 10^2^	2.8544× 10^2^	3.1832× 10^2^	2.7885× 10^2^	1.7034× 10^2^
F17	mean	1.9548× 10^3^	2.2642× 10^3^	1.9024× 10^3^	2.0244× 10^3^	2.0566× 10^3^	1.8389× 10^3^	1.9563× 10^3^	2.1257× 10^3^	2.1134× 10^3^	1.8447× 10^3^	2.4736× 10^3^	1.8893× 10^3^
	std	1.0690× 10^2^	2.1529× 10^2^	7.1183× 10^1^	1.9155× 10^2^	1.6076× 10^2^	6.1016× 10^1^	1.1669× 10^2^	1.1645× 10^2^	1.9547× 10^2^	9.9647× 10^1^	3.0046× 10^2^	7.2373× 10^1^
F18	mean	9.8052× 10^4^	5.6765× 10^4^	3.1710× 10^5^	2.6613× 10^5^	6.0203× 10^5^	2.2997× 10^5^	1.9150× 10^3^	6.6305× 10^5^	8.1518× 10^3^	2.1405× 10^5^	1.6974× 10^4^	1.2874× 10^5^
	std	9.5702× 10^4^	3.8478× 10^4^	2.2588× 10^5^	1.5736× 10^5^	3.8019× 10^5^	2.0789× 10^5^	3.2112× 10^1^	3.3742× 10^5^	9.7381× 10^3^	1.8128× 10^5^	1.5522× 10^4^	5.0433× 10^4^
F19	mean	6.7405× 10^4^	4.4112× 10^3^	9.9577× 10^3^	5.2815× 10^3^	1.7592× 10^4^	8.1353× 10^3^	1.9403× 10^3^	1.3944× 10^4^	3.3769× 10^3^	1.4321× 10^4^	7.1436× 10^3^	5.1198× 10^3^
	std	6.6497× 10^4^	2.4250× 10^3^	1.1488× 10^4^	3.7535× 10^3^	1.7264× 10^4^	8.6402× 10^3^	1.6188× 10^1^	1.1237× 10^4^	5.3192× 10^3^	1.6189× 10^4^	6.1495× 10^3^	2.3527× 10^3^
F20	mean	2.2755× 10^3^	2.5010× 10^3^	2.1971× 10^3^	2.3430× 10^3^	2.4048× 10^3^	2.2679× 10^3^	2.2467× 10^3^	2.5133× 10^3^	2.4621× 10^3^	2.1777× 10^3^	2.6998× 10^3^	2.2105× 10^3^
	std	1.0059× 10^2^	1.6603× 10^2^	8.6822× 10^1^	1.6287× 10^2^	1.5542× 10^2^	8.7595× 10^1^	1.1969× 10^2^	1.3795× 10^2^	2.1983× 10^2^	1.1596× 10^2^	1.9699× 10^2^	7.9614× 10^1^
F21	mean	2.3783× 10^3^	2.4479× 10^3^	2.3507× 10^3^	2.3556× 10^3^	2.3966× 10^3^	2.3550× 10^3^	2.3532× 10^3^	2.4475× 10^3^	2.3845× 10^3^	2.3528× 10^3^	2.4619× 10^3^	2.3355× 10^3^
	std	2.1547× 10^1^	4.2027× 10^1^	1.1255× 10^1^	1.1578× 10^1^	2.4693× 10^1^	2.7813× 10^1^	1.2784× 10^1^	1.7155× 10^1^	2.3664× 10^1^	3.3846× 10^1^	3.8648× 10^1^	9.0451× 10^0^
F22	mean	3.4928× 10^3^	2.8468× 10^3^	2.3006× 10^3^	2.4571× 10^3^	3.9296× 10^3^	2.3102× 10^3^	4.1847× 10^3^	5.9670× 10^3^	3.3583× 10^3^	2.7294× 10^3^	4.1830× 10^3^	3.4742× 10^3^
	std	2.0240× 10^3^	1.6877× 10^3^	1.3269× 10^0^	5.9712× 10^2^	1.8190× 10^3^	5.3558× 10^0^	1.8688× 10^3^	1.4867× 10^3^	1.8077× 10^3^	1.6197× 10^3^	2.3756× 10^3^	1.5783× 10^3^
F23	mean	2.7519× 10^3^	2.8423× 10^3^	2.7009× 10^3^	2.7045× 10^3^	2.7520× 10^3^	2.7135× 10^3^	2.7220× 10^3^	2.8080× 10^3^	2.7652× 10^3^	2.7198× 10^3^	2.8491× 10^3^	2.6869× 10^3^
	std	2.9881× 10^1^	7.8571× 10^1^	1.6247× 10^1^	1.7399× 10^1^	2.3409× 10^1^	2.2913× 10^1^	1.9440× 10^1^	1.9828× 10^1^	3.3474× 10^1^	4.8184× 10^1^	6.3590× 10^1^	8.4032× 10^0^
F24	mean	2.9001× 10^3^	3.0052× 10^3^	2.8690× 10^3^	2.8790× 10^3^	2.9156× 10^3^	2.8842× 10^3^	2.8926× 10^3^	2.9805× 10^3^	2.9322× 10^3^	2.8639× 10^3^	3.0385× 10^3^	2.8593× 10^3^
	std	2.6569× 10^1^	6.6312× 10^1^	1.7175× 10^1^	1.1739× 10^1^	2.6339× 10^1^	1.7191× 10^1^	2.3424× 10^1^	2.7541× 10^1^	3.6810× 10^1^	3.5838× 10^1^	7.6049× 10^1^	8.9635× 10^0^
F25	mean	2.9481× 10^3^	2.9021× 10^3^	2.8961× 10^3^	2.8873× 10^3^	2.9035× 10^3^	2.9160× 10^3^	2.8873× 10^3^	2.8931× 10^3^	2.8971× 10^3^	2.8875× 10^3^	2.8930× 10^3^	2.8868× 10^3^
	std	2.6858× 10^1^	1.9247× 10^1^	1.8302× 10^1^	2.1846× 10^0^	2.0192× 10^1^	2.1323× 10^1^	1.4172× 10^0^	1.2254× 10^1^	1.5651× 10^1^	1.3974× 10^0^	1.4364× 10^1^	1.3559× 10^0^
F26	mean	4.8285× 10^3^	5.2719× 10^3^	4.0407× 10^3^	4.0335× 10^3^	4.6365× 10^3^	3.6706× 10^3^	4.3195× 10^3^	4.8802× 10^3^	4.9149× 10^3^	3.9018× 10^3^	6.0434× 10^3^	3.9691× 10^3^
	std	5.4546× 10^2^	1.6802× 10^3^	4.7961× 10^2^	4.4870× 10^2^	3.9354× 10^2^	6.4601× 10^2^	5.1214× 10^2^	7.9276× 10^2^	3.7984× 10^2^	4.9258× 10^2^	9.7525× 10^2^	2.4801× 10^2^
F27	mean	3.2644× 10^3^	3.2740× 10^3^	3.2074× 10^3^	3.2200× 10^3^	3.2315× 10^3^	3.2342× 10^3^	3.2242× 10^3^	3.2466× 10^3^	3.2517× 10^3^	3.2011× 10^3^	3.2656× 10^3^	3.2143× 10^3^
	std	2.5752× 10^1^	3.9854× 10^1^	9.8523× 10^0^	1.0434× 10^1^	1.3605× 10^1^	1.2428× 10^1^	1.4627× 10^1^	1.5280× 10^1^	2.8241× 10^1^	9.5231× 10^0^	3.2659× 10^1^	7.4162× 10^0^
F28	mean	3.2989× 10^3^	3.2286× 10^3^	3.2185× 10^3^	3.2136× 10^3^	3.2661× 10^3^	3.2650× 10^3^	3.2191× 10^3^	3.2167× 10^3^	3.1430× 10^3^	3.2221× 10^3^	3.1347× 10^3^	3.1904× 10^3^
	std	2.0453× 10^1^	3.1479× 10^1^	2.8860× 10^1^	2.7152× 10^1^	5.7204× 10^1^	2.5751× 10^1^	2.5964× 10^1^	1.7020× 10^1^	5.3816× 10^1^	1.3912× 10^1^	5.9865× 10^1^	3.9162× 10^1^
F29	mean	3.8367× 10^3^	4.2136× 10^3^	3.5175× 10^3^	3.6672× 10^3^	3.8575× 10^3^	3.6413× 10^3^	3.7100× 10^3^	3.7783× 10^3^	3.8091× 10^3^	3.4687× 10^3^	4.0371× 10^3^	3.5198× 10^3^
	std	2.4369× 10^2^	3.4434× 10^2^	1.0040× 10^2^	2.2322× 10^2^	2.2301× 10^2^	1.0469× 10^2^	1.1610× 10^2^	1.1992× 10^2^	1.8123× 10^2^	8.5289× 10^1^	2.6906× 10^2^	7.9498× 10^1^
F30	mean	1.5774× 10^6^	1.1793× 10^4^	2.0090× 10^4^	9.0335× 10^3^	2.0499× 10^5^	1.7182× 10^4^	6.7753× 10^3^	5.8706× 10^4^	1.0583× 10^4^	1.3207× 10^5^	7.8287× 10^3^	7.8607× 10^3^
	std	1.5468× 10^6^	4.9169× 10^3^	3.7118× 10^4^	3.2975× 10^3^	2.1407× 10^5^	9.8608× 10^3^	1.3711× 10^3^	1.5394× 10^5^	3.7594× 10^3^	6.4870× 10^4^	2.0481× 10^3^	1.5336× 10^3^

**Table 4:** Results of various algorithms tested on the CEC 2017 benchmark (dim = 50).

ID	Metric	CSA	GTO	SBOA	SAO	RIME	GRO	RBMO	ED	HHWOA	IGWO	RTH	IRTH
F1	mean	1.0061× 10^8^	2.6932× 10^4^	9.8143× 10^3^	3.3774× 10^3^	4.6685× 10^6^	6.1917× 10^3^	4.3025× 10^3^	3.0661× 10^4^	6.3579× 10^3^	1.3296× 10^7^	4.9340× 10^3^	1.0207× 10^3^
	std	6.6998× 10^7^	4.0439× 10^4^	7.8747× 10^3^	4.0025× 10^3^	1.5645× 10^6^	6.6753× 10^8^	6.4880× 10^3^	3.7046× 10^4^	8.1640× 10^3^	6.8870× 10^6^	7.2141× 10^3^	7.7973× 10^2^
F3	mean	1.1318× 10^5^	2.4888× 10^4^	4.4634× 10^4^	2.3680× 10^5^	7.1933× 10^4^	1.1173× 10^5^	1.5347× 10^3^	2.2685× 10^5^	1.6399× 10^3^	3.3718× 10^4^	6.8803× 10^2^	2.3112× 10^4^
	std	1.9925× 10^4^	9.0939× 10^3^	1.0044× 10^4^	4.9013× 10^4^	1.6775× 10^4^	1.4506× 10^4^	6.6635× 10^2^	3.0384× 10^4^	1.7714× 10^3^	7.1836× 10^3^	5.3315× 10^2^	4.2277× 10^3^
F4	mean	7.2450× 10^2^	5.8271× 10^2^	5.5534× 10^2^	5.5250× 10^2^	6.2373× 10^2^	7.1388× 10^2^	5.4743× 10^2^	5.5223× 10^2^	5.2842× 10^2^	5.7996× 10^2^	4.7900× 10^2^	5.1686× 10^2^
	std	5.6940× 10^1^	5.6356× 10^1^	5.2002× 10^1^	5.3107× 10^1^	6.5492× 10^1^	9.4400× 10^1^	5.1874× 10^1^	6.8848× 10^1^	6.0806× 10^1^	4.4654× 10^1^	4.7455× 10^1^	4.3572× 10^1^
F5	mean	7.2730× 10^2^	8.3009× 10^2^	6.6839× 10^2^	6.3834× 10^2^	6.8296× 10^2^	7.0489× 10^2^	6.1652× 10^2^	8.4007× 10^2^	6.9747× 10^2^	6.5099× 10^2^	8.1089× 10^2^	5.7340× 10^2^
	std	3.5773× 10^1^	4.2449× 10^1^	3.6159× 10^1^	6.7996× 10^1^	4.5728× 10^1^	3.0326× 10^1^	2.3098× 10^1^	3.2277× 10^1^	4.2042× 10^1^	7.0291× 10^1^	3.7451× 10^1^	1.0156× 10^1^
F6	mean	6.3634× 10^2^	6.5634× 10^2^	6.0475× 10^2^	6.0045× 10^2^	6.1398× 10^2^	6.1532× 10^2^	6.0074× 10^2^	6.2338× 10^2^	6.1949× 10^2^	6.0235× 10^2^	6.5042× 10^2^	6.0017× 10^2^
	std	5.1760× 10^0^	8.0573× 10^0^	2.3656× 10^0^	3.7724× 10^-1^	5.3362× 10^0^	4.3506× 10^0^	4.3140× 10^-1^	5.5694× 10^0^	7.8254× 10^0^	1.0683× 10^0^	6.5773× 10^0^	5.8131× 10^-2^
F7	mean	1.2148× 10^3^	1.4666× 10^3^	9.9864× 10^2^	1.1730× 10^3^	9.9296× 10^2^	1.0137× 10^3^	8.8313× 10^2^	1.1366× 10^3^	1.1452× 10^3^	9.7105× 10^2^	1.4861× 10^3^	8.3252× 10^2^
	std	7.4495× 10^1^	1.2384× 10^2^	7.9150× 10^1^	5.6625× 10^1^	6.6777× 10^1^	6.6363× 10^1^	2.6777× 10^1^	3.3048× 10^1^	9.3219× 10^1^	9.8242× 10^1^	1.1143× 10^2^	1.9228× 10^1^
F8	mean	9.9059× 10^2^	1.1498× 10^3^	9.6129× 10^2^	9.3514× 10^2^	9.9077× 10^2^	1.0042× 10^3^	9.2585× 10^2^	1.1285× 10^3^	1.0010× 10^3^	9.3885× 10^2^	1.1299× 10^3^	8.6822× 10^2^
	std	3.1994× 10^1^	4.1361× 10^1^	3.5935× 10^1^	6.4712× 10^1^	3.9415× 10^1^	4.1073× 10^1^	2.8497× 10^1^	2.9867× 10^1^	4.5153× 10^1^	4.7389× 10^1^	4.6002× 10^1^	1.2269× 10^1^
F9	mean	5.5287× 10^3^	1.1214× 10^3^	2.3187× 10^3^	9.6698× 10^3^	5.3978× 10^3^	3.8155× 10^3^	1.0987× 10^3^	9.8008× 10^3^	3.0773× 10^3^	1.4590× 10^3^	1.1068× 10^3^	9.5177× 10^3^
	std	1.4208× 10^3^	1.6148× 10^3^	6.6211× 10^2^	1.0880× 10^2^	2.3403× 10^3^	1.5112× 10^3^	2.3884× 10^2^	4.1579× 10^3^	9.2371× 10^2^	6.6414× 10^2^	1.3211× 10^3^	3.4327× 10^1^
F10	mean	8.4274× 10^3^	9.3183× 10^3^	6.5729× 10^3^	7.0743× 10^3^	7.2573× 10^3^	7.5917× 10^3^	8.0732× 10^3^	8.8150× 10^3^	7.7771× 10^3^	1.2411× 10^4^	8.0583× 10^3^	7.3729× 10^3^
	std	1.5206× 10^3^	1.9628× 10^3^	7.7841× 10^2^	2.1516× 10^3^	9.1081× 10^2^	6.7791× 10^2^	8.7808× 10^2^	4.7207× 10^2^	8.3962× 10^2^	3.3366× 10^3^	1.0747× 10^3^	6.9001× 10^2^
F11	mean	2.2089× 10^3^	1.3014× 10^3^	1.2605× 10^3^	1.4837× 10^3^	1.5163× 10^3^	2.0220× 10^3^	1.2534× 10^3^	1.6398× 10^3^	1.3536× 10^3^	1.4213× 10^3^	1.3374× 10^3^	1.2134× 10^3^
	std	3.3316× 10^2^	3.7047× 10^1^	4.8919× 10^1^	2.4635× 10^2^	7.3619× 10^1^	5.0661× 10^2^	4.1089× 10^1^	1.5333× 10^2^	6.4632× 10^1^	7.1821× 10^1^	8.7637× 10^1^	2.4017× 10^1^
F12	mean	1.9042× 10^8^	4.3600× 10^6^	3.4325× 10^6^	4.0049× 10^6^	8.1233× 10^7^	1.2137× 10^7^	5.3393× 10^5^	3.5426× 10^6^	8.7679× 10^5^	2.3543× 10^7^	2.1962× 10^5^	1.1081× 10^6^
	std	1.1068× 10^8^	3.8771× 10^6^	2.5735× 10^6^	2.4617× 10^6^	5.3087× 10^7^	6.0742× 10^6^	4.0476× 10^5^	3.0959× 10^6^	4.8006× 10^5^	1.4855× 10^7^	1.2838× 10^5^	4.4008× 10^5^
F13	mean	1.1326× 10^5^	1.5210× 10^3^	1.2579× 10^3^	7.4823× 10^3^	2.0486× 10^5^	8.2442× 10^3^	1.4485× 10^3^	9.4118× 10^3^	8.7782× 10^3^	3.8757× 10^5^	8.6025× 10^3^	8.3578× 10^3^
	std	8.6611× 10^4^	1.1100× 10^4^	1.1711× 10^4^	7.6736× 10^3^	8.8566× 10^4^	3.5245× 10^3^	1.2197× 10^4^	7.6332× 10^3^	8.0889× 10^3^	1.6366× 10^5^	8.6463× 10^3^	6.2844× 10^3^
F14	mean	1.0699× 10^5^	2.9181× 10^4^	1.4552× 10^5^	7.6592× 10^4^	2.3580× 10^5^	1.9410× 10^5^	1.5742× 10^3^	3.9817× 10^5^	8.3630× 10^3^	5.9020× 10^4^	6.2833× 10^3^	5.3734× 10^4^
	std	8.4716× 10^4^	2.5792× 10^4^	8.3258× 10^4^	5.3496× 10^4^	1.4515× 10^5^	1.3863× 10^5^	3.0708× 10^1^	2.1319× 10^5^	6.2333× 10^3^	4.8849× 10^4^	3.5226× 10^3^	2.3916× 10^4^
F15	mean	5.0313× 10^4^	1.6094× 10^4^	1.1361× 10^4^	1.2034× 10^4^	6.0197× 10^4^	1.0276× 10^4^	9.1573× 10^3^	1.3718× 10^4^	1.2448× 10^4^	9.8461× 10^4^	9.6313× 10^3^	5.2293× 10^3^
	std	2.8826× 10^4^	8.6353× 10^3^	7.2988× 10^3^	6.1921× 10^3^	3.7033× 10^4^	5.8115× 10^3^	1.0277× 10^4^	1.0212× 10^4^	9.0788× 10^3^	4.6299× 10^4^	8.0729× 10^3^	3.0271× 10^3^
F16	mean	3.2291× 10^3^	3.5500× 10^3^	2.6084× 10^3^	3.1167× 10^3^	3.5700× 10^3^	2.6998× 10^3^	3.1046× 10^3^	4.0171× 10^3^	3.4757× 10^3^	2.7538× 10^3^	3.7384× 10^3^	2.8895× 10^3^
	std	4.6609× 10^2^	4.3251× 10^2^	3.7668× 10^2^	4.3147× 10^2^	3.4989× 10^2^	2.7874× 10^2^	3.4405× 10^2^	3.0363× 10^2^	4.6972× 10^2^	7.1569× 10^2^	4.3891× 10^2^	3.4816× 10^2^
F17	mean	2.9195× 10^3^	3.4006× 10^3^	2.6491× 10^3^	2.6760× 10^3^	3.2685× 10^3^	2.6841× 10^3^	2.8503× 10^3^	3.2643× 10^3^	3.1381× 10^3^	2.6349× 10^3^	3.4887× 10^3^	2.6727× 10^3^
	std	2.7226× 10^2^	3.2628× 10^2^	2.5667× 10^2^	3.1011× 10^2^	3.7153× 10^2^	2.2467× 10^2^	2.8814× 10^2^	3.1314× 10^2^	3.1099× 10^2^	5.9631× 10^2^	4.0167× 10^2^	2.2049× 10^2^
F18	mean	7.7975× 10^5^	1.5975× 10^5^	1.7484× 10^6^	1.2684× 10^6^	3.1366× 10^6^	1.4659× 10^6^	2.5464× 10^3^	3.5409× 10^6^	3.7819× 10^6^	9.6780× 10^5^	4.1765× 10^6^	8.5722× 10^5^
	std	5.8282× 10^5^	9.5450× 10^4^	9.3749× 10^5^	1.6958× 10^6^	2.1994× 10^6^	7.4807× 10^5^	2.9827× 10^2^	2.3298× 10^6^	3.4000× 10^4^	7.4831× 10^5^	2.9673× 10^4^	3.5905× 10^5^
F19	mean	9.1656× 10^5^	1.7667× 10^4^	1.7495× 10^4^	1.9516× 10^4^	4.9398× 10^4^	2.0995× 10^4^	6.8977× 10^3^	9.6275× 10^3^	1.6169× 10^4^	5.4529× 10^4^	1.3777× 10^4^	1.2172× 10^4^
	std	7.8390× 10^5^	1.0998× 10^4^	1.3175× 10^4^	1.0569× 10^4^	3.2077× 10^4^	1.0397× 10^4^	1.1055× 10^4^	1.0207× 10^4^	1.1239× 10^4^	2.7900× 10^4^	1.0042× 10^4^	8.2462× 10^3^
F20	mean	2.8859× 10^3^	3.1936× 10^3^	2.6903× 10^3^	2.8369× 10^3^	3.1126× 10^3^	2.7344× 10^3^	2.9847× 10^3^	3.4999× 10^3^	3.1199× 10^3^	2.8880× 10^3^	3.3303× 10^3^	2.8381× 10^3^
	std	3.3196× 10^2^	3.2576× 10^2^	2.8019× 10^2^	3.1167× 10^2^	2.6189× 10^2^	1.9139× 10^2^	2.1919× 10^2^	1.3929× 10^2^	3.0706× 10^2^	5.0422× 10^2^	2.8285× 10^2^	1.9769× 10^2^
F21	mean	2.5010× 10^3^	2.6350× 10^3^	2.4226× 10^3^	2.4262× 10^3^	2.4864× 10^3^	2.4658× 10^3^	2.4300× 10^3^	2.6484× 10^3^	2.4952× 10^3^	2.4187× 10^3^	2.6376× 10^3^	2.3748× 10^3^
	std	2.6116× 10^1^	6.0906× 10^1^	2.5396× 10^1^	2.1516× 10^1^	4.2529× 10^1^	3.0487× 10^1^	3.1347× 10^1^	3.1080× 10^1^	4.1563× 10^1^	2.4425× 10^1^	6.0141× 10^1^	1.6028× 10^1^
F22	mean	9.8196× 10^3^	1.1038× 10^4^	7.0325× 10^3^	7.8648× 10^3^	9.1403× 10^3^	7.4484× 10^3^	9.4121× 10^3^	1.1086× 10^4^	9.5720× 10^3^	1.2659× 10^4^	9.9843× 10^3^	8.9756× 10^3^
	std	1.9645× 10^3^	1.2783× 10^3^	2.5282× 10^3^	2.4530× 10^3^	1.0792× 10^3^	3.1062× 10^3^	1.7929× 10^3^	4.5495× 10^2^	1.1309× 10^3^	4.0602× 10^3^	8.3250× 10^2^	5.7891× 10^2^
F23	mean	3.0373× 10^3^	3.1887× 10^3^	2.8625× 10^3^	2.8521× 10^3^	2.9503× 10^3^	2.9276× 10^3^	2.9452× 10^3^	3.1068× 10^3^	3.0225× 10^3^	2.8881× 10^3^	3.2158× 10^3^	2.8315× 10^3^
	std	5.9087× 10^1^	8.9178× 10^1^	3.6448× 10^1^	2.9978× 10^1^	4.7530× 10^1^	3.0110× 10^1^	6.3324× 10^1^	3.7490× 10^1^	5.3944× 10^1^	9.7407× 10^1^	1.1214× 10^2^	1.8742× 10^1^
F24	mean	3.1596× 10^3^	3.3775× 10^3^	3.0159× 10^3^	3.0341× 10^3^	3.1170× 10^3^	3.0896× 10^3^	3.1221× 10^3^	3.3132× 10^3^	3.1757× 10^3^	3.0293× 10^3^	3.3767× 10^3^	2.9901× 10^3^
	std	6.5399× 10^1^	1.1499× 10^2^	3.2357× 10^1^	8.2431× 10^1^	5.1140× 10^1^	3.2318× 10^1^	6.2483× 10^1^	6.2619× 10^1^	6.8836× 10^1^	9.0477× 10^1^	1.1748× 10^2^	1.3382× 10^1^
F25	mean	3.2827× 10^3^	3.1116× 10^3^	3.0896× 10^3^	3.0399× 10^3^	3.1040× 10^3^	3.2833× 10^3^	3.0701× 10^3^	3.0838× 10^3^	3.0522× 10^3^	3.0886× 10^3^	3.0560× 10^3^	3.0398× 10^3^
	std	7.6004× 10^1^	2.4692× 10^1^	2.3398× 10^1^	2.8291× 10^1^	4.3321× 10^1^	7.6243× 10^1^	2.6078× 10^1^	3.3028× 10^1^	3.7189× 10^1^	3.1530× 10^1^	4.1819× 10^1^	2.9817× 10^1^
F26	mean	7.3727× 10^3^	8.4232× 10^3^	5.3702× 10^3^	4.9564× 10^3^	5.7954× 10^3^	5.5915× 10^3^	5.6434× 10^3^	7.1172× 10^3^	7.2445× 10^3^	5.2327× 10^3^	8.6120× 10^3^	4.6721× 10^3^
	std	9.2851× 10^2^	3.1097× 10^3^	1.5448× 10^3^	2.0330× 10^2^	6.7797× 10^2^	1.2721× 10^3^	5.1427× 10^2^	3.3136× 10^2^	9.8059× 10^2^	7.5562× 10^2^	2.6897× 10^3^	1.7545× 10^2^
F27	mean	3.7627× 10^3^	3.7985× 10^3^	3.3206× 10^3^	3.3398× 10^3^	3.4973× 10^3^	3.5686× 10^3^	3.4251× 10^3^	3.7948× 10^3^	3.6474× 10^3^	3.2972× 10^3^	3.6495× 10^3^	3.3554× 10^3^
	std	1.6583× 10^2^	1.8379× 10^2^	4.0717× 10^1^	6.4461× 10^1^	7.4864× 10^1^	7.5700× 10^1^	1.2615× 10^2^	1.2441× 10^2^	1.4977× 10^2^	4.0092× 10^1^	1.3327× 10^2^	6.0297× 10^1^
F28	mean	3.7798× 10^3^	3.3638× 10^3^	3.3493× 10^3^	3.2945× 10^3^	3.3612× 10^3^	3.6432× 10^3^	3.3358× 10^3^	3.3841× 10^3^	3.3132× 10^3^	3.3481× 10^3^	3.3020× 10^3^	3.3046× 10^3^
	std	1.3824× 10^2^	4.9948× 10^1^	3.5706× 10^1^	2.4935× 10^1^	3.1899× 10^1^	1.0693× 10^2^	2.8017× 10^1^	3.0300× 10^1^	2.4819× 10^1^	3.9802× 10^1^	3.8078× 10^1^	1.2283× 10^1^
F29	mean	5.1170× 10^3^	5.1110× 10^3^	3.7409× 10^3^	3.8490× 10^3^	4.6146× 10^3^	4.1729× 10^3^	4.3188× 10^3^	4.8887× 10^3^	4.6566× 10^3^	3.7495× 10^3^	4.7027× 10^3^	3.8929× 10^3^
	std	4.5475× 10^2^	6.6936× 10^2^	2.2728× 10^2^	3.2789× 10^2^	4.1467× 10^2^	2.3171× 10^2^	2.9683× 10^2^	4.4946× 10^2^	3.7499× 10^2^	1.5925× 10^2^	4.2710× 10^2^	2.1670× 10^2^
F30	mean	9.4168× 10^7^	1.2771× 10^6^	9.5028× 10^5^	9.4314× 10^5^	2.5384× 10^7^	1.4581× 10^6^	1.8047× 10^6^	2.8306× 10^6^	1.0559× 10^6^	8.3887× 10^6^	7.9462× 10^5^	8.9353× 10^5^
	std	2.8810× 10^7^	4.3268× 10^5^	1.7820× 10^5^	1.6497× 10^5^	1.1156× 10^7^	3.1136× 10^5^	1.0419× 10^6^	1.0731× 10^6^	3.5146× 10^5^	2.3153× 10^6^	1.4337× 10^5^	1.1944× 10^5^

**Table 5:** Results of various algorithms tested on the CEC 2017 benchmark (dim = 100).

ID	Metric	CSA	GTO	SBOA	SAO	RIME	GRO	RBMO	ED	HHWOA	IGWO	RTH	IRTH
F1	mean	1.0796× 10^10^	1.2291× 10^8^	1.9079× 10^8^	1.4125× 10^8^	9.2498× 10^7^	2.8043× 10^10^	3.2114× 10^7^	3.5563× 10^8^	6.2511× 10^3^	8.4984× 10^9^	4.8468× 10^3^	2.9204× 10^5^
	std	2.3153× 10^9^	1.1691× 10^8^	2.8426× 10^8^	9.5248× 10^7^	2.0914× 10^7^	8.3110× 10^9^	1.7226× 10^7^	2.4699× 10^8^	7.5622× 10^3^	3.6494× 10^9^	5.8996× 10^3^	2.4211× 10^5^
F3	mean	3.6575× 10^5^	1.6701× 10^5^	2.4996× 10^5^	7.3205× 10^5^	4.9513× 10^5^	3.1793× 10^5^	8.1763× 10^4^	6.2053× 10^5^	3.0502× 10^5^	2.6815× 10^5^	6.6119× 10^4^	2.1178× 10^5^
	std	3.6090× 10^4^	2.0188× 10^4^	2.4605× 10^4^	1.5136× 10^5^	6.4844× 10^4^	2.2025× 10^4^	1.4554× 10^4^	8.1622× 10^4^	4.4695× 10^4^	3.4550× 10^4^	1.7946× 10^4^	1.6732× 10^4^
F4	mean	2.2867× 10^3^	1.0400× 10^3^	9.2686× 10^2^	7.5589× 10^2^	9.1987× 10^2^	3.2238× 10^3^	8.8382× 10^2^	1.0617× 10^3^	7.1074× 10^2^	1.4502× 10^3^	6.7215× 10^2^	7.0039× 10^2^
	std	3.6033× 10^2^	1.6256× 10^2^	8.1214× 10^1^	6.4034× 10^1^	9.7811× 10^1^	5.4772× 10^2^	6.2345× 10^1^	9.8521× 10^1^	4.3889× 10^1^	3.0432× 10^2^	4.3372× 10^1^	4.9982× 10^1^
F5	mean	1.2132× 10^3^	1.3331× 10^3^	9.9814× 10^2^	1.1473× 10^3^	1.0565× 10^3^	1.1903× 10^3^	9.5088× 10^2^	1.5208× 10^3^	1.0952× 10^3^	9.6144× 10^2^	1.2924× 10^3^	7.3373× 10^2^
	std	5.9548× 10^1^	5.2029× 10^1^	8.8253× 10^1^	2.1584× 10^2^	9.2563× 10^1^	6.0460× 10^1^	8.1137× 10^1^	7.7780× 10^1^	9.1349× 10^1^	1.0623× 10^2^	6.8410× 10^1^	3.2536× 10^1^
F6	mean	6.5615× 10^2^	6.6147× 10^2^	6.2481× 10^2^	6.1112× 10^2^	6.3541× 10^2^	6.4021× 10^2^	6.1012× 10^2^	6.5117× 10^2^	6.3956× 10^2^	6.1177× 10^2^	6.5239× 10^2^	6.0383× 10^2^
	std	3.4967× 10^0^	3.6748× 10^0^	7.3954× 10^0^	2.2631× 10^0^	5.2757× 10^0^	5.2722× 10^0^	2.7333× 10^0^	2.5228× 10^0^	5.4985× 10^0^	2.3041× 10^0^	3.4803× 10^0^	7.0743× 10^-1^
F7	mean	2.6702× 10^3^	2.9435× 10^3^	1.9067× 10^3^	1.9674× 10^3^	1.6975× 10^3^	2.1054× 10^3^	1.3340× 10^3^	2.6006× 10^3^	2.2952× 10^3^	1.4707× 10^3^	2.9192× 10^3^	1.1375× 10^3^
	std	1.9015× 10^2^	1.8111× 10^2^	1.8453× 10^2^	6.8935× 10^1^	1.6381× 10^2^	1.8256× 10^2^	1.1242× 10^2^	1.9477× 10^2^	2.1745× 10^2^	1.3233× 10^2^	1.6237× 10^2^	3.6184× 10^1^
F8	mean	1.5488× 10^3^	1.7576× 10^3^	1.2899× 10^3^	1.3881× 10^3^	1.3501× 10^3^	1.4761× 10^3^	1.2193× 10^3^	1.8030× 10^3^	1.4177× 10^3^	1.2549× 10^3^	1.6695× 10^3^	1.0453× 10^3^
	std	7.6411× 10^1^	8.0124× 10^1^	7.3924× 10^1^	2.5748× 10^2^	8.7527× 10^1^	7.5646× 10^1^	7.5834× 10^1^	7.1840× 10^1^	8.3247× 10^1^	1.1857× 10^2^	8.4022× 10^1^	2.7592× 10^1^
F9	mean	2.1637× 10^4^	2.3710× 10^4^	1.7973× 10^4^	9.3598× 10^3^	2.7913× 10^4^	2.1153× 10^4^	6.1673× 10^3^	5.2880× 10^4^	1.3210× 10^4^	1.7740× 10^4^	2.2005× 10^4^	2.9671× 10^3^
	std	2.8353× 10^3^	1.4288× 10^3^	3.5589× 10^3^	4.7432× 10^3^	1.1286× 10^4^	3.7656× 10^3^	2.3224× 10^3^	7.9331× 10^3^	3.3169× 10^3^	8.3005× 10^3^	1.5265× 10^3^	5.4116× 10^2^
F10	mean	1.8640× 10^4^	1.6711× 10^4^	1.5033× 10^4^	2.1979× 10^4^	1.7178× 10^4^	1.9026× 10^4^	1.9198× 10^4^	2.3004× 10^4^	1.6596× 10^4^	2.6118× 10^4^	1.5900× 10^4^	1.6966× 10^4^
	std	1.6322× 10^3^	2.9862× 10^3^	1.5073× 10^3^	7.9384× 10^3^	1.4149× 10^3^	1.5011× 10^3^	1.7005× 10^3^	7.1066× 10^2^	1.4348× 10^3^	7.4900× 10^3^	1.4060× 10^3^	1.3518× 10^3^
F11	mean	5.2436× 10^4^	6.8503× 10^3^	1.2879× 10^4^	1.3678× 10^5^	7.2701× 10^3^	5.2460× 10^4^	3.2691× 10^3^	7.6291× 10^4^	2.3262× 10^3^	1.2675× 10^4^	2.2341× 10^3^	3.6329× 10^3^
	std	1.2015× 10^4^	2.0028× 10^3^	4.5184× 10^3^	3.5820× 10^3^	1.1316× 10^3^	9.9105× 10^3^	3.3443× 10^2^	1.2533× 10^3^	2.3293× 10^2^	4.4250× 10^3^	2.4816× 10^2^	5.5426× 10^2^
F12	mean	1.4599× 10^9^	8.6200× 10^7^	5.5383× 10^7^	4.3425× 10^7^	7.0047× 10^8^	1.3882× 10^9^	2.6548× 10^7^	6.6270× 10^7^	7.7935× 10^6^	4.2442× 10^8^	2.5147× 10^6^	7.4138× 10^6^
	std	4.2559× 10^8^	7.5239× 10^7^	3.4302× 10^7^	2.2567× 10^7^	2.7708× 10^8^	8.8712× 10^8^	1.7397× 10^7^	2.8065× 10^7^	4.0611E+06	1.7948× 10^8^	1.2313× 10^6^	2.9190× 10^6^
F13	mean	5.7871× 10^4^	2.0412× 10^4^	1.2586× 10^4^	5.0653× 10^3^	3.0502× 10^5^	4.6621× 10^5^	1.1527× 10^4^	1.0654× 10^4^	1.2346× 10^4^	4.6021× 10^5^	9.7754× 10^3^	4.2581× 10^3^
	std	2.1425× 10^4^	8.0092× 10^3^	7.6554× 10^3^	2.5408× 10^3^	9.2779× 10^4^	5.0084× 10^5^	1.3190× 10^4^	6.8587× 10^3^	4.6171× 10^3^	2.5097× 10^5^	6.2239× 10^3^	1.8084× 10^3^
F14	mean	1.8320× 10^6^	3.4519× 10^5^	1.5068× 10^6^	7.4591× 10^5^	4.2998× 10^6^	2.0630× 10^6^	2.3440× 10^3^	4.4042× 10^6^	9.4906× 10^4^	1.2201× 10^6^	5.6723× 10^4^	9.7278× 10^5^
	std	1.1244× 10^6^	1.4883× 10^5^	8.1152× 10^5^	3.8218× 10^5^	2.3267× 10^6^	6.6870× 10^5^	4.3042× 10^2^	2.3672× 10^6^	3.6996× 10^4^	6.5436× 10^5^	2.8685× 10^4^	3.5057× 10^5^
F15	mean	4.7306× 10^4^	1.0331× 10^4^	9.3792× 10^3^	3.4138× 10^3^	1.4111× 10^5^	8.4546× 10^3^	5.9420× 10^3^	5.4500× 10^3^	4.8994× 10^3^	1.5791× 10^5^	6.3513× 10^3^	3.5015× 10^3^
	std	2.2130× 10^4^	6.7643× 10^3^	1.9862× 10^4^	1.8422× 10^3^	5.0047× 10^4^	3.1098× 10^3^	2.8072× 10^3^	4.1157× 10^3^	2.7186× 10^3^	9.8522× 10^4^	6.9279× 10^3^	1.1830× 10^3^
F16	mean	7.0208× 10^3^	6.4016× 10^3^	4.8320× 10^3^	5.3092× 10^3^	6.7794× 10^3^	5.7443× 10^3^	6.1604× 10^3^	8.8893× 10^3^	5.6002× 10^3^	4.7585× 10^3^	5.8946× 10^3^	5.2741× 10^3^
	std	6.3029× 10^2^	7.1431× 10^2^	6.0708× 10^2^	1.1795× 10^3^	6.9960× 10^2^	7.4077× 10^2^	5.4160× 10^2^	1.1174× 10^3^	7.7195× 10^2^	5.5010× 10^2^	7.1371× 10^2^	5.1970× 10^2^
F17	mean	5.4594× 10^3^	5.9235× 10^3^	4.5471× 10^3^	4.9120× 10^3^	5.4187× 10^3^	4.4947× 10^3^	5.1200× 10^3^	6.1932× 10^3^	5.3864× 10^3^	4.5835× 10^3^	5.8588× 10^3^	4.5123× 10^3^
	std	5.6769× 10^2^	7.9792× 10^2^	6.5705× 10^2^	9.3993× 10^2^	6.6691× 10^2^	3.8943× 10^2^	6.2444× 10^2^	7.5057× 10^2^	6.6045× 10^2^	1.2004× 10^3^	5.9552× 10^2^	4.4271× 10^2^
F18	mean	2.6621× 10^6^	6.7322× 10^5^	2.5007× 10^6^	3.5943× 10^6^	6.5375× 10^6^	4.1983× 10^6^	9.3063× 10^4^	1.6759× 10^7^	2.9216× 10^5^	3.0857× 10^6^	2.2075× 10^5^	1.4884× 10^6^
	std	1.6010× 10^6^	2.6362× 10^5^	1.2516× 10^6^	2.2512× 10^6^	3.1007× 10^6^	1.8357× 10^6^	4.1975× 10^4^	1.1618× 10^7^	1.4517× 10^5^	1.6189× 10^6^	1.1547× 10^5^	5.6961× 10^5^
F19	mean	2.0478× 10^7^	7.3673× 10^3^	8.8842× 10^3^	5.8296× 10^3^	8.2060× 10^6^	1.2716× 10^4^	8.7776× 10^3^	5.9345× 10^3^	8.2243× 10^3^	3.3839× 10^5^	7.4914× 10^3^	3.8244× 10^3^
	std	1.5773× 10^7^	4.9003× 10^3^	1.6854× 10^4^	4.6241× 10^3^	5.7651× 10^6^	1.1651× 10^4^	9.2107× 10^3^	5.2469× 10^3^	8.5088× 10^3^	2.3844× 10^5^	5.8117× 10^3^	1.3403× 10^3^
F20	mean	4.8156× 10^3^	5.5846× 10^3^	4.2361× 10^3^	5.5287× 10^3^	5.6094× 10^3^	4.6611× 10^3^	4.9284× 10^3^	6.6569× 10^3^	5.2328× 10^3^	5.2528× 10^3^	5.4210× 10^3^	4.6769× 10^3^
	std	5.1783× 10^2^	5.7573× 10^2^	6.9821× 10^2^	1.6169× 10^3^	5.4963× 10^2^	4.5768× 10^2^	4.5800× 10^2^	3.4571× 10^2^	4.7387× 10^2^	1.3730× 10^3^	4.3822× 10^2^	5.4930× 10^2^
F21	mean	3.0769× 10^3^	3.3189× 10^3^	2.7472× 10^3^	2.8097× 10^3^	2.9303× 10^3^	2.9238× 10^3^	2.8678× 10^3^	3.3408× 10^3^	2.9789× 10^3^	2.7703× 10^3^	3.3226× 10^3^	2.5931× 10^3^
	std	9.6224× 10^1^	1.2900× 10^2^	6.6601× 10^1^	1.5486× 10^2^	9.5086× 10^1^	5.7827× 10^1^	9.2293× 10^1^	9.9453× 10^1^	1.2854× 10^2^	1.1572× 10^2^	1.7464× 10^2^	3.3507× 10^1^
F22	mean	2.3682× 10^4^	2.2456× 10^4^	1.7810× 10^4^	1.8420× 10^4^	1.9373× 10^4^	2.1876× 10^4^	2.2099× 10^4^	2.4743× 10^4^	1.8637× 10^4^	2.9514× 10^4^	1.9858× 10^4^	1.9522× 10^4^
	std	2.2739× 10^3^	3.1970× 10^3^	3.2386× 10^3^	4.4716× 10^3^	1.5509× 10^3^	3.0638× 10^3^	2.4624× 10^3^	3.6785× 10^2^	1.4238× 10^3^	7.5541× 10^3^	1.1098× 10^3^	1.2455× 10^3^
F23	mean	3.7322× 10^3^	4.0449× 10^3^	3.2214× 10^3^	3.1615× 10^3^	3.4100× 10^3^	3.5780× 10^3^	3.5774× 10^3^	3.9007× 10^3^	3.6484× 10^3^	3.2099× 10^3^	3.7893× 10^3^	3.1388× 10^3^
	std	1.2203× 10^2^	2.5004× 10^2^	6.3055× 10^1^	5.1478× 10^1^	6.3659× 10^1^	5.9218× 10^1^	1.1621× 10^2^	1.3628× 10^2^	1.4742× 10^2^	5.4358× 10^1^	1.3995× 10^2^	4.3529× 10^1^
F24	mean	4.6520× 10^3^	5.0088× 10^3^	3.8244× 10^3^	3.6369× 10^3^	4.0183× 10^3^	4.2925× 10^3^	4.1980× 10^3^	4.5563× 10^3^	4.4495× 10^3^	3.6930× 10^3^	4.6006× 10^3^	3.6110× 10^3^
	std	2.2888× 10^2^	5.3163× 10^2^	1.0782× 10^2^	8.4064× 10^1^	1.1986× 10^2^	1.0559× 10^2^	1.4439× 10^2^	2.3528× 10^2^	2.7535× 10^2^	7.9090× 10^1^	1.9475× 10^2^	4.3077× 10^1^
F25	mean	4.9747× 10^3^	3.7091× 10^3^	3.6117× 10^3^	3.4974× 10^3^	3.6249× 10^3^	5.1695× 10^3^	3.5389× 10^3^	3.7553× 10^3^	3.3525× 10^3^	4.1318× 10^3^	3.3077× 10^3^	3.3562× 10^3^
	std	3.9930× 10^2^	9.7696× 10^1^	7.4014× 10^1^	3.9488× 10^1^	9.2461× 10^1^	5.0022× 10^2^	6.2828× 10^1^	1.0524× 10^2^	6.7634× 10^1^	1.9647× 10^2^	5.9860× 10^1^	5.1087× 10^1^
F26	mean	1.9919× 10^4^	2.3920× 10^4^	1.4278× 10^4^	9.4952× 10^3^	1.3588× 10^4^	2.0100× 10^4^	1.3091× 10^4^	1.8071× 10^4^	1.7686× 10^4^	1.0887× 10^4^	2.1498× 10^4^	9.0624× 10^3^
	std	1.8088× 10^3^	3.2193× 10^3^	3.1031× 10^3^	5.0358× 10^2^	1.3213× 10^3^	3.3593× 10^3^	1.9563× 10^3^	1.6297× 10^3^	1.5716× 10^3^	7.6587× 10^2^	2.2914× 10^3^	5.5218× 10^2^
F27	mean	4.2327× 10^3^	4.1328× 10^3^	3.5945× 10^3^	3.4367× 10^3^	3.8055× 10^3^	4.0934× 10^3^	3.5445× 10^3^	4.0157× 10^3^	3.9980× 10^3^	3.5194× 10^3^	3.7098× 10^3^	3.5263× 10^3^
	std	2.4959× 10^2^	4.1808× 10^2^	7.7135× 10^1^	4.9131× 10^1^	1.3087× 10^2^	1.4688× 10^2^	7.7158× 10^1^	2.4917× 10^2^	2.4679× 10^2^	4.7968× 10^1^	1.2183× 10^2^	3.9839× 10^1^
F28	mean	5.6824× 10^3^	3.7775× 10^3^	3.7433× 10^3^	3.5264× 10^3^	3.7073× 10^3^	6.8902× 10^3^	3.7673× 10^3^	4.3062× 10^3^	3.4623× 10^3^	4.5759× 10^3^	3.4018× 10^3^	3.4917× 10^3^
	std	4.9773× 10^2^	9.3473× 10^1^	6.0429× 10^1^	3.9822× 10^1^	6.0461× 10^1^	8.5430× 10^2^	1.3487× 10^2^	4.1938× 10^2^	3.7518× 10^1^	4.0310× 10^2^	3.6614× 10^1^	2.4332× 10^1^
F29	mean	1.0688× 10^4^	8.2739× 10^3^	6.1100× 10^3^	6.0089× 10^3^	8.5881× 10^3^	7.3525× 10^3^	7.3305× 10^3^	8.1931× 10^3^	7.4736× 10^3^	6.2400× 10^3^	7.3982× 10^3^	6.4274× 10^3^
	std	1.1410× 10^3^	6.4737× 10^2^	6.7163× 10^2^	5.0757× 10^2^	7.9917× 10^2^	4.7817× 10^2^	6.1574× 10^2^	1.4097× 10^3^	6.4783× 10^2^	5.4218× 10^2^	5.7776× 10^2^	3.1264× 10^2^
F30	mean	2.5154× 10^8^	4.1555× 10^5^	6.0889× 10^4^	3.3083× 10^4^	7.2816× 10^7^	3.9971× 10^6^	5.1329× 10^4^	1.3432× 10^6^	2.8723× 10^4^	1.2941× 10^7^	1.2044× 10^4^	1.2884× 10^4^
	std	1.0039× 10^8^	2.2547× 10^5^	6.8850× 10^4^	2.0488× 10^4^	3.1741× 10^7^	3.0652× 10^6^	5.2548× 10^4^	1.3423× 10^6^	3.1480× 10^4^	6.6092× 10^6^	4.7693× 10^3^	2.7861× 10^3^

**Table 6:** Results for various algorithms on the CEC 2017.

Statistical Results	CSA	GTO	SBOA	SAO	RIME	GRO	RBMO	ED	HHWOA	IGWO	RTH
Dim = 30 (+/=/−)	30/0/0	27/1/2	27/3/0	28/2/0	30/0/0	29/1/0	21/3/6	29/1/0	22/4/4	25/5/0	24/0/6
Dim = 50 (+/=/−)	30/0/0	28/0/2	25/4/1	27/3/0	30/0/0	28/2/0	25/2/3	30/0/0	25/2/3	27/1/2	22/4/4
Dim = 100 (+/=/−)	30/0/0	27/0/3	25/5/0	27/2/1	30/0/0	30/0/0	26/1/3	30/0/0	24/2/4	25/5/0	22/5/3

**Table 7:** Friedman mean rank test results.

Suites	CEC2017					
Dimensions	30		50		100	
Algorithms	M.R	T.R	M.R	T.R	M.R	T.R
CSA	9.17	12	9.55	12	9.72	12
GTO	8.44	9	8.93	10	8.62	10
SBOA	4.76	3	4.34	3	4.58	3
SAO	5.00	4	4.34	3	4.58	3
RIME	9.03	11	8.10	9	7.79	8
GRO	6.72	7	7.13	8	8.55	9
RBMO	3.44	2	4.27	2	4.48	2
ED	8.93	10	9.44	11	9.65	11
HHWOA	6.00	6	6.13	6	5.31	5
IGWO	5.79	5	6.03	5	6.48	7
RTH	7.89	8	7.00	7	5.68	6
IRTH	2.75	1	2.68	1	2.51	1

**Table 8:** Experimental results of path planning for each algorithm in scenario 1.

Algorithm	CSA	GTO	SBOA	SAO	RIME	GRO	RBMO	ED	HHWOA	IGWO	RTH	IRTH
mean	419.61	416.11	415.17	415.19	419.99	416.63	435.07	416.94	416.05	535.52	419.16	414.26
median	418.42	415.63	414.98	415.09	419.39	417.10	428.35	416.71	415.58	533.47	417.74	414.91
max	445.69	421.51	421.69	416.88	435.52	419.93	475.40	422.28	428.34	589.77	439.95	415.40
min	415.66	408.92	408.00	414.44	414.64	411.06	414.99	415.53	414.39	485.18	416.23	409.81

**Table 9:** Experimental results of path planning for each algorithm in scenario 2.

Algorithm	CSA	GTO	SBOA	SAO	RIME	GRO	RBMO	ED	HHWOA	IGWO	RTH	IRTH
mean	418.74	414.98	415.29	414.82	419.21	417.17	431.96	417.19	415.50	543.70	418.03	413.16
median	417.97	414.96	415.05	414.75	418.74	417.19	430.74	417.11	415.60	542.89	417.68	414.57
max	426.49	421.63	417.93	415.97	425.32	419.16	455.78	418.86	417.06	593.32	421.11	415.21
min	415.20	410.62	414.22	413.89	416.03	411.58	416.33	415.46	414.07	504.76	416.66	409.11

**Table 10:** Experimental results of path planning for each algorithm in scenario 3.

Algorithm	CSA	GTO	SBOA	SAO	RIME	GRO	RBMO	ED	HHWOA	IGWO	RTH	IRTH
mean	448.58	443.14	432.96	435.98	453.42	437.32	Inf	435.80	434.59	Inf	439.48	426.47
median	444.99	438.96	434.45	434.07	455.38	436.99	467.35	435.69	433.48	627.88	436.34	426.56
max	482.86	493.82	438.50	455.64	476.76	441.13	Inf	440.04	458.43	Inf	488.07	432.37
min	436.95	433.27	424.53	424.87	433.35	434.38	440.05	432.85	431.87	549.64	426.46	415.65

**Table 11:** Experimental results of path planning for each algorithm in scenario 4.

Algorithm	CSA	GTO	SBOA	SAO	RIME	GRO	RBMO	ED	HHWOA	IGWO	RTH	IRTH
mean	532.25	497.29	Inf	Inf	464.62	443.95	Inf	442.58	484.80	Inf	500.90	436.81
median	523.86	506.76	453.80	468.00	462.77	443.32	Inf	441.07	476.71	Inf	500.81	437.33
max	658.77	553.79	Inf	Inf	520.56	450.93	Inf	458.01	647.34	Inf	608.92	450.47
min	447.32	435.26	418.55	417.41	426.01	439.00	464.20	428.98	414.55	532.06	433.89	415.08

## Data Availability

Data are contained within the article.
